# Enhanced and Stable Carbon Capture through Hierarchical Sorbent and Process Optimization Using Biochar from Deoiled Cake

**DOI:** 10.1021/acsomega.6c03610

**Published:** 2026-06-29

**Authors:** Sai Krishna Reddy Velagala, Arohi Pore, Ylias Sabri, Rajarathinam Parthasarathy, Inkollu Sreedhar

**Affiliations:** † Department of Chemical Engineering, 209298 BITS Pilani Hyderabad Campus, Hyderabad 50078, India; ‡ Centre of Advanced Materials and Industrial Chemistry, School of Sciences, 5376 RMIT University, Melbourne, Victoria 3001, Australia; § Department of Chemical and Environmental Engineering, School of Engineering, RMIT University, Melbourne, Victoria 3001, Australia

## Abstract

In this study, a novel functionalized biochar derived from deoiled cake biomass was developed using an integrated multiparameter optimization framework for effective carbon dioxide (CO_2_) captured under a simulated flue gas mixture. Unlike conventional synthesis that focus on individual parameters, this work systematically integrates various process variables within a single framework to establish a clear structure–property–performance relationship. The results demonstrate the synthesis pathways and chemical activation conditions play a decisive role in tuning the key physicochemical properties of the adsorbent, which directly influence CO_2_ capture efficiency. Statistical optimization was carried out using response surface methodology (RSM). Under optimized conditions of 30 °C, 200 mL/min, 3 g, and 180 min, a maximum CO_2_ uptake of 4.9 mmol/g was achieved. Among the investigated variables, adsorbent amount and contact time were identified as the most influential factors, whereas temperature and flow rate showed comparatively weaker effects. Kinetic analysis confirms that the pseudo-second order (PSO) model provided the best fit with an *R* ^2^ of 0.98 and a rate constant (*k*_2_) of 0.092 g mmol^–1^ h^–1^, indicating that the adsorption process involves a combination of physisorption and chemisorption with strong adsorbate–surface interactions. The adsorption isotherm data were well described by the Langmuir model with an *R* ^2^ of 0.99, indicating monolayer adsorption behavior. The isosteric heat of adsorption, around 40.02 kJ/mol, further supports strong CO_2_ adsorbent interactions. In addition, the adsorbent exhibited stable cyclic performance over 20 adsorption–desorption cycles with marginal capacity loss, demonstrating its potential for practical flue gas CO_2_ capture applications.

## Graphical abstract

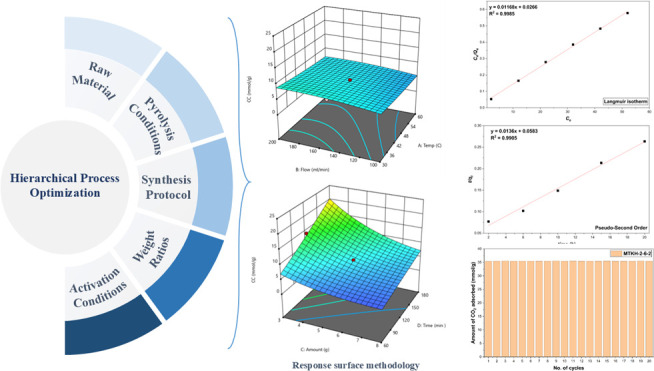

## 1 Introduction

Carbon dioxide (CO_2_) is the primary greenhouse gas (GHG) associated with human activities, particularly the combustion of fossil fuels, and is a major contributor to global climate change. The continuous increase in atmospheric CO_2_ levels has led to severe environmental consequences, including rising temperatures, extreme weather events, glacier melting, and sea-level rise.^1^ According to the International Energy Agency (IEA), global CO_2_ emissions increased by approximately 0.4% in 2025, reaching a record high of nearly 38.4 gigatonnes (Gt), marking the highest level recorded until date.^2^ It is crucial to reduce CO_2_ emissions, prompting extensive global efforts to develop efficient, cost-effective adsorbents for carbon capture.^3^ Among various adsorbent classes, activated carbon (AC) stands out due to its high surface area, tunable porosity, thermal stability, hydrophobicity, low regeneration energy, abundance, and low cost.^4^ Despite these advantages, pristine biochar often exhibits limited CO_2_ capture performance, primarily due to insufficient surface area, minimal microporosity, and a lack of reactive surface functionalities.^5^ These limitations are strongly governed by the nature of the biomass precursor and the pyrolysis conditions employed. Consequently, the careful selection of feedstock along with the optimized pyrolysis conditions is crucial for developing biochar with enhanced textural properties suitable for CO_2_ adsorption. Even with optimized biochar, the affinity toward CO_2_, especially at low concentrations relevant to direct air capture and flue gas conditions, remains inadequate. Enhancing the surface chemistry and porosity of biochar through chemical activation and functionalization has emerged as an effective strategy to address these challenges.^6^

Activation using chemical agents, such as ZnCl_2_, H_3_PO_4_, H_2_O, O_2_, CO_2_, NaOH, and KOH, plays a pivotal role in tailoring the porosity and surface area of carbon materials.^7,8^ Among these, KOH is particularly effective in generating high surface areas and microporosity, however its highly corrosive nature presents practical and operational challenges.^9^ Alternatively, activating agents such as K_2_CO_3_ and K_2_C_2_O_4_ have gained increasing attention because they can provide comparable activation efficiency with lower corrosiveness, reduced cost, and relatively environmentally benign operation.^10^ In addition to pore development, surface functionalization using nitrogen-rich precursors such as urea, thiourea, and melamine can further enhance the basicity and nucleophilic character of the carbon surface, thereby promoting stronger Lewis acid–base interactions with acidic CO_2_ molecules. Such modifications may also improve the isosteric heat of adsorption, contributing to enhanced adsorption performance over a broader temperature range.^11,12^

Previous studies have demonstrated the potential of combining chemical activation and nitrogen functionalization to enhance CO_2_ adsorption performance. For instance, KOH-activated biochar derived from wood chips exhibited enhanced surface area and microporosity, achieving a CO_2_ adsorption capacity of 4.05 mmol/g under simulated flue gas conditions.^13^ Similarly, urea-modified activated carbon derived from coconut shell showed enhanced nitrogen content, improved porosity, and strong CO_2_ affinity, with an adsorption capacity of 5.0 mmol/g.^14^ Likewise, various N-functionalized and activated carbons derived from different biomass sources such as bamboo shoot shell, peanut shell, corn straw, lentil shell, and olive pomace have also demonstrated promising CO_2_ capture performance, with adsorption capacities ranging from 0.90 to 3.76 mmol/g.^15–19^ Although these studies clearly demonstrate the beneficial effects of activation and surface functionalization, most investigations have primarily focused on optimizing individual parameters independently rather than systematically evaluating their combined influence on CO_2_ capture performance. Consequently, a comprehensive understanding of how factors such as precursor type, pyrolysis conditions, synthesis route, weight ratios, and activation parameters collectively govern the structure–property–performance relationship remains limited, particularly under low-concentration conditions relevant to practical flue gas applications.

To address these gaps, the present study focuses on the development of a novel biochar-based adsorbent derived from deoiled cake biomass through an integrated multiparameter optimization framework. Unlike conventional approaches, this study systematically integrates precursor selection, pyrolysis conditions, synthesis route, weight ratios, and activation parameters within a single framework to evaluate their collective influence on the physicochemical properties of the developed adsorbent and the resulting CO_2_ capture performance, thereby providing deeper insight into the structure–property–performance relationship governing CO_2_ adsorption. In addition, process parameters were optimized using response surface methodology (RSM) based on a central composite design (CCD), enabling a systematic evaluation of the influence of critical operating variables on CO_2_ capture behavior. To the best of our knowledge, such an integrated multiparameter optimization strategy has not been reported either for deoiled cake-derived adsorbents or more broadly for carbon-based sorbents. Adsorption experiments were conducted in a fixed-bed reactor under a simulated flue gas mixture (CO_2_/O_2_/N_2_: 12:8:80 vol %), and detailed investigations including adsorption kinetics, isotherms, and thermodynamic analyses were performed to elucidate the adsorption mechanism. Furthermore, cyclic adsorption–desorption studies were carried out to evaluate the stability and practical applicability of the developed adsorbent.

## 2 Materials and Methods

### 2.1 Materials and Characterization Tools

The materials used in this study include thiourea (CH_4_N_2_S-99%, extrapure AR) and potassium oxalate monohydrate (K_2_C_2_O_4_·H_2_O-99%), which were procured from SRL Pvt. Ltd., Maharashtra, India. All the deoiled cake biomass precursors were purchased from the local market in Hyderabad, India.

### 2.2 Preparation of Adsorbent

The complete optimization procedure is provided in the Supporting Information (Section S1) and Figure 1, while the final optimized synthesis route is described as follows. Mustard deoiled cake biomass was initially washed, oven-dried at 110 °C overnight and sieved to obtain a particle size of 150–200 μm. The prepared biomass was then pyrolyzed at 600 °C for 2 h and allowed to cool naturally to obtain the biochar. Subsequently, the obtained biochar was mixed with thiourea in a 1:1 weight ratio and thermally treated at 500 °C for 1 h with a heating rate of 5 °C/min. The resulting material was washed with deionized water to remove unreacted species, followed by overnight drying at 110 °C. The dried sample was then impregnated with K_2_C_2_O_4_ in a 1:3 weight ratio and stirred for 12 h, followed by filtration. The impregnated material was subsequently activated at 600 °C for 2 h with a heating rate of 5 °C/min. Finally, the activated product was washed sequentially with 0.1 M HCl and deionized water until a neutral pH was attained, followed by drying in a vacuum oven at 110 °C overnight for further use.

**Figure 1:**
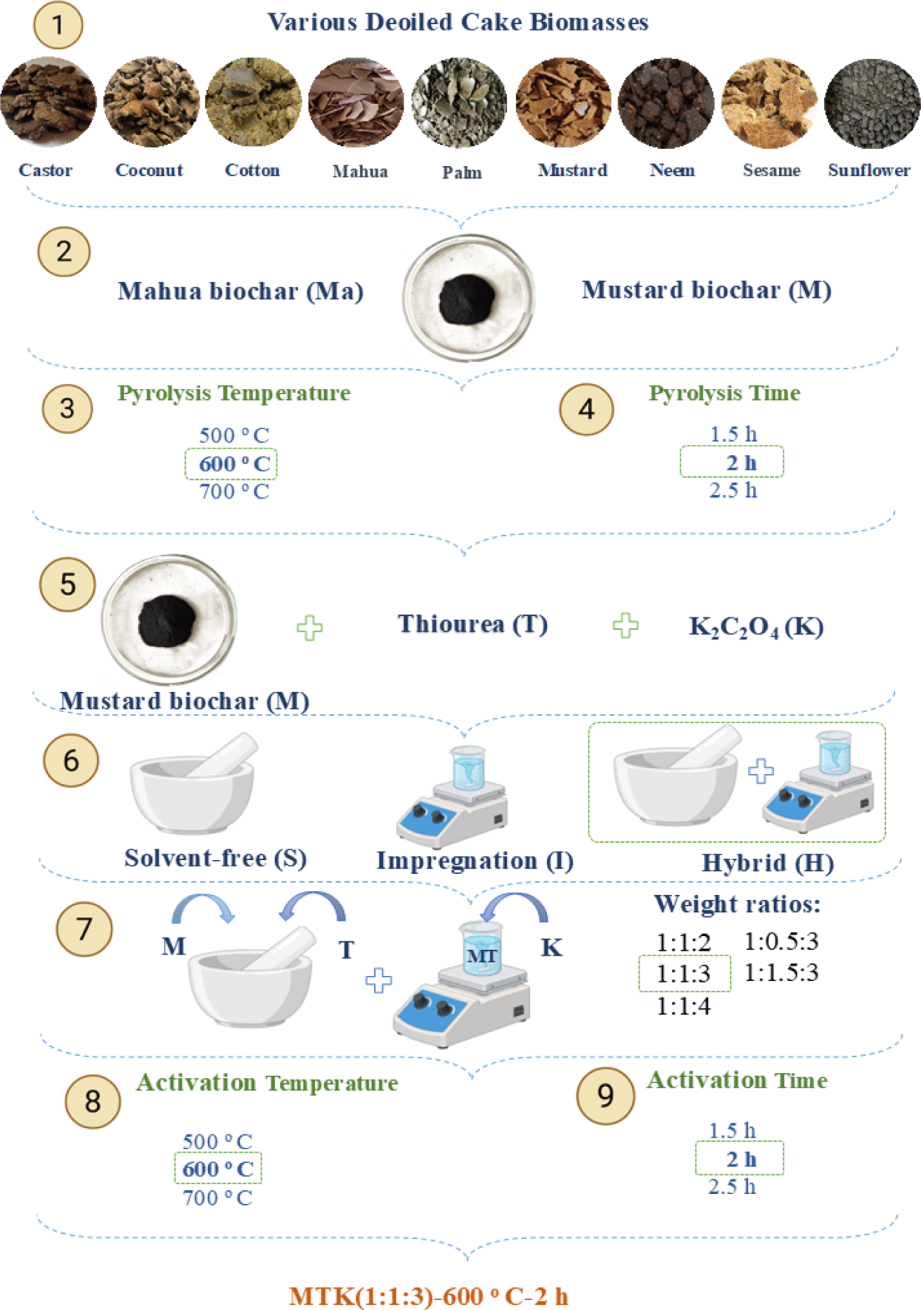
Overview of synthesis protocol.

### 2.3 Experimental Setup

The experimental setup was designed to investigate the adsorption process, as illustrated in Figure 2. An adsorption column containing a stationary bed served as the central component of this system, playing a key role in the adsorption process. To achieve a stable and controlled temperature, an electric heating unit was incorporated into the system. Gas lines were connected to the system to ensure a continuous flow of gases required for adsorption. Mass flow controllers were used for the precise adjustment of gas flow rates (range: 10–200 mL/min), facilitating accurate regulation of gas delivery. The system temperature was continuously monitored using a thermocouple to ensure operation within the desired range. The inlet of the quartz tube was connected to the gas lines, functioning as a fixed-bed adsorber. Inside the quartz tube, quartz wool acted as a supportive medium for the adsorbent, effectively preventing the loss of adsorbent particles into the gas flow during the reaction. After passing through the adsorption column, the outlet gas was cooled and subsequently analyzed using a gas analyzer. This analysis enabled a detailed evaluation of the gas composition and was essential for determining the CO_2_ concentration in the exhaust stream and assessing the adsorption efficiency.

**Figure 2:**
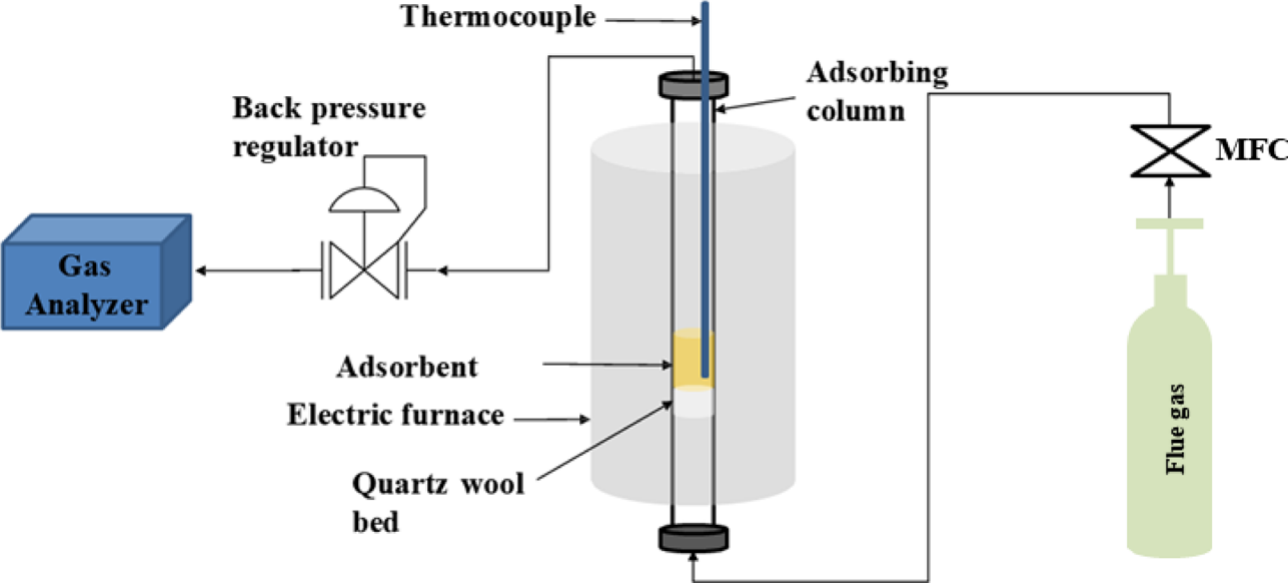
Experimental reactor setup.

### 2.4 Characterization

Various characterization techniques were utilized for comprehensive material analysis. The Brunauer–Emmett–Teller (BET) method was employed to evaluate surface area and porosity, using a Microtrac BEL SORP mini II instrument controlled by BEL SORP mini software. The morphological and microstructural characteristics of the samples were investigated through field emission scanning electron microscopy (FE-SEM) with an FEI Apreo LoVac microscope that features a Retractable STEM 3+ detector and a DBS detector, while elemental composition and mapping were executed with an integrated Aztec standard energy dispersive X-ray spectroscopy (EDX) system. X-ray diffraction (XRD) was performed with an ULTIMA-IV model that utilizes a Cu Kα radiation source and a dTex detector. Raman spectroscopy was conducted using a Renishaw InVia Reflex confocal Raman spectrophotometer equipped with a 532 nm laser to assess the structural ordering and defect characteristics of the carbon materials. The elemental composition was quantified with a CHNSO elemental analyzer (PerkinElmer 2400 Series II), allowing for the measurement of heteroatom incorporation. Fourier Transform Infrared (FTIR) spectroscopy was carried out using a Jasco FTIR-4200 spectrometer operating between 600 and 4000 cm^–1^ with a resolution of ±0.5 cm^–1^ and a wavenumber accuracy of ±0.01 cm^–1^, and included a Ge/KBr beam splitter, DLATGS detector, and DRA-81 accessory. Lastly, the surface chemical composition and bonding states were analyzed through X-ray photoelectron spectroscopy (XPS) using a Thermo Scientific K-Alpha system, which features a monochromatic Al Kα X-ray source, offering in-depth insights into surface functional groups and elemental states.

## 3 Results and Discussion

### 3.1 Physico-Chemical Attributes

#### 3.1.1 Textural and Morphological Properties

The N_2_ adsorption–desorption isotherms obtained at 77 K for both pristine and modified carbon samples reveal distinct difference in textural characteristics as shown in Figure 3. According to the IUPAC classification, all four samples exhibit characteristic Type I adsorption isotherm with a slight hysteresis loop, indicating the presence of a predominantly microporous structure with a minor contribution from mesoporosity.^20^ The sharp uptake observed at low relative pressure is particularly significant for CO_2_ adsorption, because narrow micropores provide stronger adsorption potential through enhanced adsorbent–adsorbate interactions, especially under low CO_2_ partial pressure conditions relevant to flue gas capture. This observation is consistent with IUPAC guidelines, where Type I isotherms are typically associated with microporous solids and rapid filling of narrow micropores at low relative pressures.^21^ The observed porosity in M-6-2 primarily originates from the dehydrogenation and devolatilization of lignocellulosic components during pyrolysis, which facilitates the formation of initial pore networks within the carbon matrix.^22^ However, in the absence of chemical activation, pore development remains limited, resulting in a comparatively lower specific surface area (463 m^2^/g) and total pore volume (0.30 cm^3^/g).

**Figure 3:**
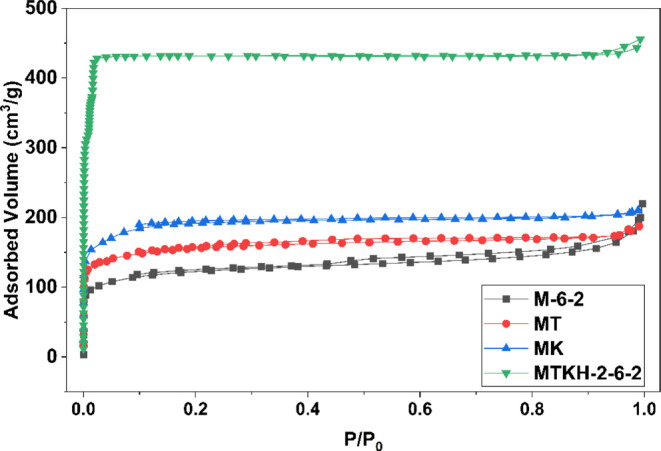
N_2_ adsorption–desorption isotherm at 77 K.

In contrast, MK and MTKH-2-6-2 exhibit significantly more developed pore structures than MT and M-6-2, indicating a predominantly microporous framework with hierarchical characteristics, as summarized in Table 1. The enhanced porosity is primarily attributed to the activating effect of K_2_C_2_O_4_, which decomposes into K_2_CO_3_, CO, and CO_2_, thereby facilitating chemical etching of the carbon framework. The in situ generated K_2_CO_3_ further reacts with carbon to produce metallic potassium, which intercalates into the carbon lattice and induces structural expansion and pore formation. These observations are in good agreement with previous reports on potassium-based activating agents. Nazir et al.^23^ reported that K_2_C_2_O_4_ first decomposes to K_2_CO_3_, which subsequently reacts with carbon through gasification, progressively etching the carbon framework and generating micropores. Similarly, Sevilla et al.^24^ observed that potassium oxalate activation strongly favors micropore development, while hierarchical porosity can emerge under optimized activation conditions. Furthermore, the intercalation of K^+^ species into the carbon lattice has been reported to induce lattice expansion at lower temperatures, whereas pore widening becomes more dominant at elevated temperatures due to intensified carbon–potassium interactions. The enhanced pore development of MK and MTKH-2-6-2 observed in this study aligns well with these reported mechanisms.

**Table 1:** Textural Properties of Pristine and Modified Carbon at 77 K

s. no	name of the sample	CO_2_ uptake^a^ (mmol/g)	*S*_BET_ ^b^ (m^2^/g)	*V*_t_ ^c^ (cm^3^/g)	*V*_micro_ ^d^ (cm^3^/g)	*V*_meso_ ^e^ (cm^3^/g)
1	M-6-2	0.71	463	0.30	0.18	0.12
2	MT	0.82	559	0.32	0.21	0.11
3	MK	0.85	756	0.37	0.29	0.08
4	MTKH-2-6-2	1.56	2996	0.69	0.62	0.07

a Screening conditions: 30 °C, 100 mL/min, 3 g, 60 min.

b Surface area was calculated using the BET method at *P*/*P*_0_ = 0.0–0.1.

c Total pore volume at *P*/*P*_0_ = 0.99.

d Total micropore volume evaluated by the *t*-plot method.

e Total mesopore volume evaluated by the BJH-plot method.

Meanwhile, the thermal decomposition of thiourea produces gaseous species such as NH_3_, HNCS, and CS_2_, which predominantly contribute to the incorporation of N and S functionalities onto the carbon surface rather than substantial pore generation. As a result, MT exhibits only a marginal increase in surface area relative to M-6-2, whereas MK demonstrates a considerably higher surface area owing to effective chemical activation by K_2_C_2_O_4_. These results confirm that chemical activation primarily governs pore development, while thiourea treatment mainly modifies the surface chemistry of the carbon framework. In the case of MTKH-2-6-2, the heteroatom-rich defect sites introduced through thiourea treatment act as anchoring sites for potassium species during activation, thereby promoting more effective lattice expansion and carbon etching. Such synergistic behavior is consistent with previous reports on N/S codoped carbons subjected to potassium-based activation.^25^ As a result, MTKH-2-6-2 exhibits substantial improvement in surface area, total pore volume, and micropore volume compared to the individual treatments alone, while simultaneously retaining the N and S surface functionalities introduced by thiourea, demonstrating that the combined approach achieves both enhanced porosity and enriched surface chemistry in a single material.

Figure 4 presents the pore size distribution obtained using the NLDFT method, where all samples exhibit a predominance of pores below 2 nm, confirming their microporous nature. Among the investigated samples, MTKH-2-6-2 displays a sharp and intense micropore peak centered at ∼1 nm along with a minor mesoporous contribution, indicating a predominantly microporous framework with hierarchical characteristics. Such a pore architecture is highly favorable for CO_2_ adsorption because narrow micropores provide strong adsorbent–adsorbate interactions under low CO_2_ partial pressure conditions, while the limited mesopore fraction facilitates molecular diffusion and improves accessibility to the adsorption sites. The progressive enhancement in microporosity from M-6-2 to MT, MK, and MTKH-2-6-2 correlates well with the observed increase in CO_2_ uptake, indicating that the superior adsorption performance of MTKH-2-6-2 is primarily governed by its highly developed microporous structure.^26^ These observations are consistent with previous studies reporting that narrow micropores are the dominant contributors to CO_2_ uptake at low pressure, whereas mesopores mainly assist in diffusion and transport within interconnected porous networks.^27^

**Figure 4:**
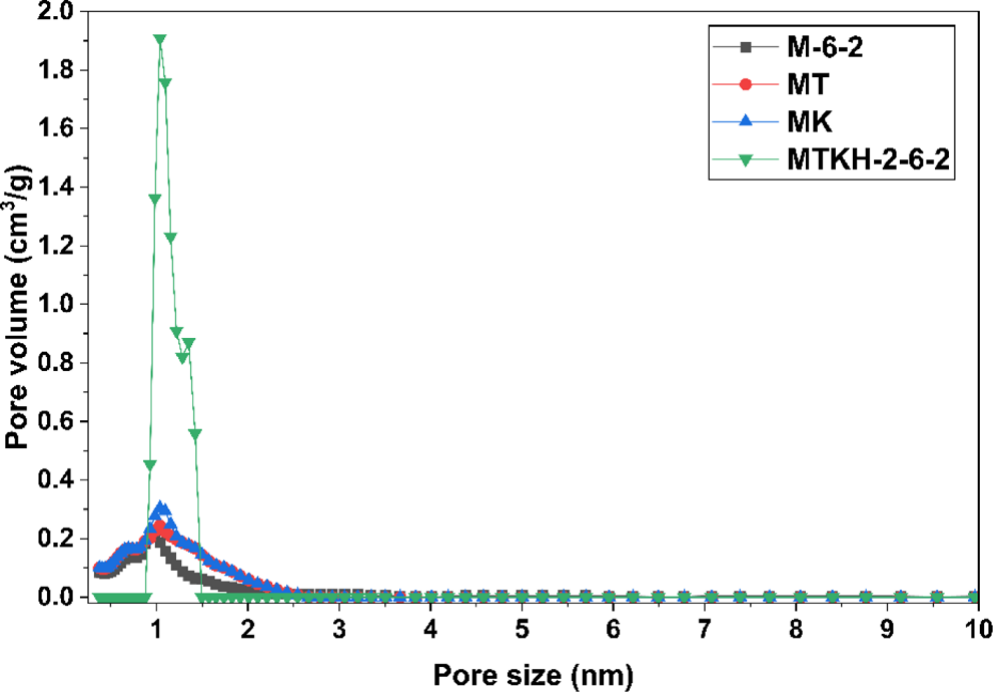
Pore size distribution based on the NLDFT method.

The SEM micrographs shown in Figure 5 further supports the textural analysis. M-6-2 exhibits a relatively compact and dense morphology with limited surface roughness, consistent with its lower surface area and pore volume.^28^ Upon thiourea modification, MT displays slight surface fragmentation and increased roughness, indicating surface functionalization without significant pore development. In contrast, MK exhibits a more open and honeycomb-like porous morphology, highlighting the strong influence of K_2_C_2_O_4_ activation on pore development.^16^ Among all samples, MTKH-2-6-2 shows the most developed porous structure, characterized by sponge-like channels and well-defined perforations distributed throughout the carbon framework. Such morphological evolution enhances the accessibility of CO_2_ molecules to the internal adsorption sites, thereby contributing to the superior adsorption performance of MTKH-2-6-2. Similar transitions from dense carbon structures to highly porous and interconnected networks following chemical activation have also been reported in studies by Lim et al. and Tang et al.,^29,30^ where potassium-based activating agents promoted extensive pore development and enhanced surface accessibility, thereby improving gas diffusion and adsorption performance.

**Figure 5:**
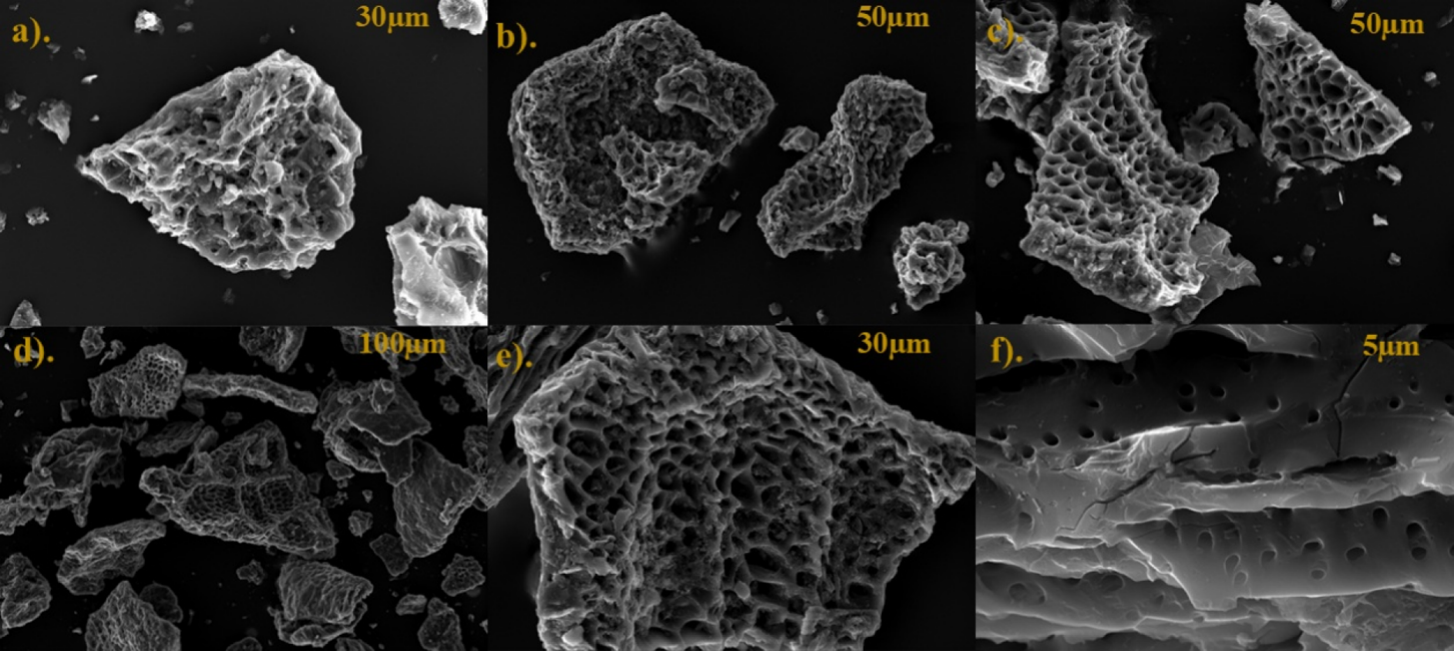
SEM micrographs of (a). M-6-2, (b). MT, (c). MK, and (d–f). MTKH-2-6-2.

#### 3.1.2 Structural Characteristics

The structural characteristics of the developed carbon materials were investigated using XRD and Raman spectroscopy, as shown in Figures 6 and 7. The XRD patterns of all samples exhibit two broad diffraction peaks centered at approximately 2θ = 25.4° and 43.1°, corresponding to the (002) and (100) planes, respectively, indicating a predominantly amorphous carbon structure with low graphitic ordering.^15^ Such broad diffraction features are commonly observed in biomass-derived activated carbons, where the amorphous nature is generally associated with a higher density of defects and active sites favorable for gas adsorption.^31^ Raman spectra further reveal the structural disorder of the carbon materials through two characteristic bands observed at 1355 cm^–1^ and 1583 cm^–1^, corresponding to the D and G bands of disordered and graphitic carbon, respectively. These bands are in good agreement with the findings reported by Sun et al. and Gui et al.^32,33^ for cellulose and corncob shell derived heteroatom-doped biochar’s, where these bands were associated with defect formation and variations in graphitic ordering within the carbon framework. The gradual increase in the *I*_D_/*I*_G_ ratio from 0.88 for M-6-2 to 0.89 for MT and 0.91 for MK indicates the progressive introduction of structural defects following chemical modification. Among all samples, MTKH-2-6-2 exhibits the highest *I*_D_/*I*_G_ value of 0.95, suggesting that the combined effect of heteroatom incorporation and K_2_C_2_O_4_ activation induces greater lattice distortion and defect generation within the carbon structure.

**Figure 6:**
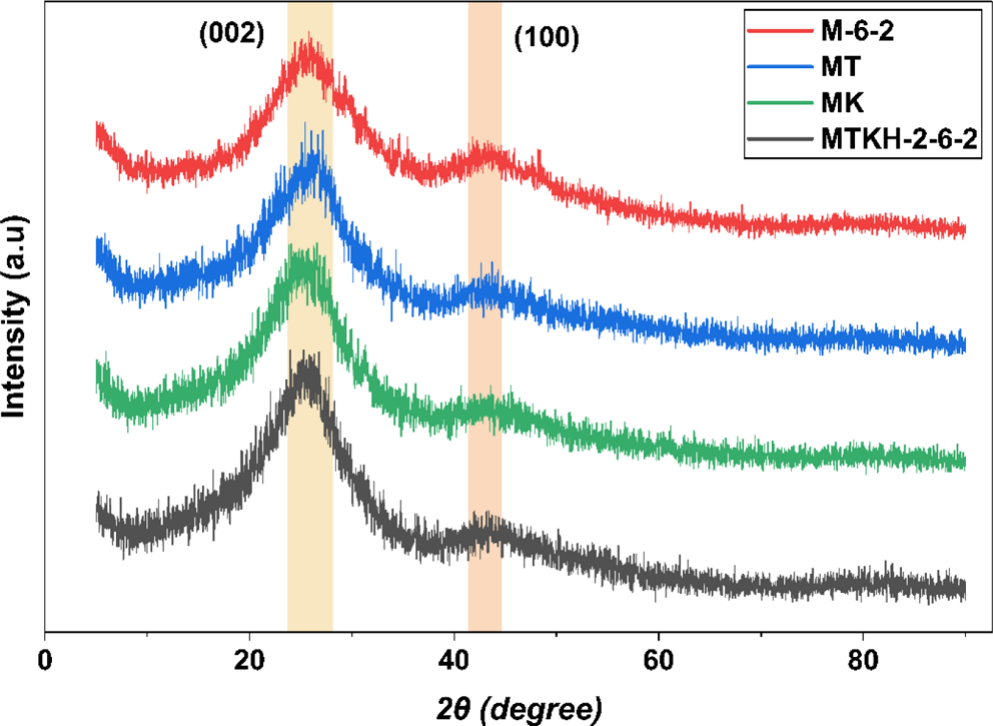
X-ray diffraction spectra for structural analysis.

**Figure 7:**
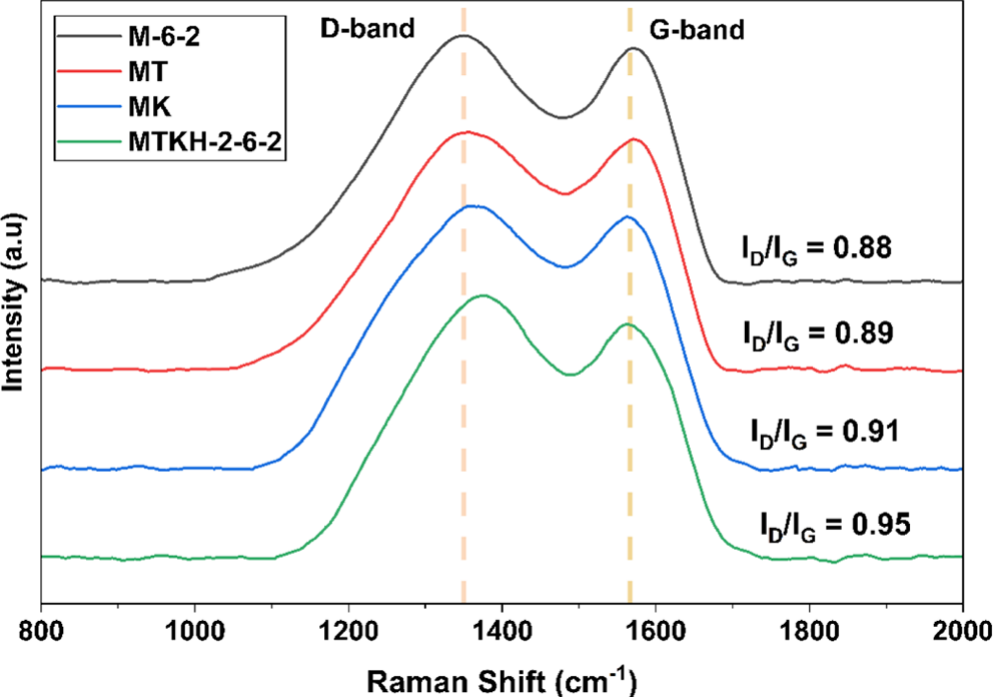
Defective analysis by Raman spectra.

The incorporation of N and S functionalities together with K_2_C_2_O_4_ activation promotes the formation of vacancies, edge defects, and distorted carbon domains, thereby increasing the overall defect density of the material. Such defect-rich structures can provide additional adsorption sites and enhance surface reactivity, facilitating stronger interactions between CO_2_ molecules and the carbon surface.^36^ This increased structural disorder is also consistent with the textural properties discussed earlier, where higher defect density correlates with enhanced pore development. Consequently, MTKH-2-6-2 exhibited the highest CO_2_ uptake of 1.59 mmol/g under the initial screening conditions among all investigated samples, indicating a strong correlation between structural disorder, enhanced pore development, and improved adsorption performance.

#### 3.1.3 Chemical Composition and Surface Functional Properties

In addition to the structural characteristics, the chemical composition of the adsorbents was analyzed using CHNSO elemental analysis, and the corresponding results are summarized in Table 2. The results reveal noticeable variations in the weight percentages of C, H, N, S, and O among the four samples, highlighting the significant influence of chemical modification on the surface chemistry of the developed carbons. Among all samples, MT exhibits the highest heteroatom enrichment, with N and S contents of 16.2 and 4.01 wt %, respectively, confirming the successful incorporation of thiourea-derived functionalities despite the relatively lower treatment temperature.^34^ Following activation, MTKH-2-6-2 shows a slight reduction in heteroatom content, with N and S contents of 13.2 and 3.53 wt %, respectively, suggesting that the combined effect of thiourea treatment and chemical activation enables substantial heteroatom retention while simultaneously promoting pore development. The enhanced nitrogen content is particularly beneficial for CO_2_ adsorption because nitrogen-containing functionalities introduce Lewis basic sites that can interact strongly with acidic CO_2_ molecules, thereby improving adsorption affinity. Similar observations were reported by Zhang et al.^4^ and Li et al.^35^ where urea-derived nitrogen incorporation improved the CO_2_ adsorption performance of carbon materials by increasing the availability of surface-active sites and strengthening interactions with CO_2_ molecules.

**Table 2:** CHNSO Elemental Composition and Atomic Ratios of Pristine and Modified Biochar

		CHNSO (elemental percentage-wt %)					atomic ratio	
s. no	name of the adsorbent	C	H	N	S	O	H/C	O/C
1	M-2-6	66.79	2.16	5.29	0.21	9.22	0.38	0.10
2	MT	61.22	1.89	16.2	4.01	8.54	0.39	0.10
3	MK	61.93	1.91	4.89	0.19	8.88	0.41	0.08
4	MTKH-2-6-2	62.07	1.42	13.2	3.53	7.89	0.27	0.09

In contrast, M-6-2 and MK exhibit comparatively lower N and S contents, which mainly originate from the naturally occurring heteroatoms present in the biomass precursor, since the absence of thiourea treatment limits additional heteroatom incorporation. The relatively similar heteroatom content observed for these two samples further suggests that K_2_C_2_O_4_ activation primarily contributes to pore development rather than substantial modification of surface chemistry.^31^ With respect to oxygen content, no significant variation is observed among the samples, as reflected by the comparable O/C atomic ratios, indicating similar surface polarity across the developed materials. Although oxygen-containing surface groups may contribute to CO_2_ adsorption through dipole–quadrupole interactions, their influence is generally less pronounced than that of nitrogen functionalities. Conversely, the H/C atomic ratio decreases progressively for the chemically modified samples, particularly for MTKH-2-6-2, indicating a higher degree of carbonization and aromaticity compared to the pristine biochar.^36^ Lower H/C ratios are commonly associated with more condensed and structurally stable carbon frameworks, which can contribute to improved adsorption stability.

The FTIR spectra displays several distinct peaks that correspond to the stretching and bending vibrations of surface functional groups, as illustrated in Figure 8. The broad band around 3310 cm^–1^ is attributed to O–H and N–H stretching vibrations, originating from hydroxyl groups naturally present in the biochar matrix together with amine functionalities introduced through thiourea treatment. The comparatively higher intensity of this band in MT and MTKH-2-6-2 confirms the successful incorporation of nitrogen-containing groups following surface modification. Similar features have been reported for nitrogen-doped biochars, where such functionalities contribute to improved CO_2_ affinity through enhanced surface interactions.^36^ A distinct peak observed at 1674 cm^–1^ corresponds to C=O stretching vibrations associated with carbonyl or amide-type functionalities. The increased intensity of this peak in thiourea-modified samples suggests the formation of nitrogen-associated carbonyl structures during thermal treatment, which can promote stronger interactions with CO_2_ molecules through dipole–quadrupole interactions.^37^

**Figure 8:**
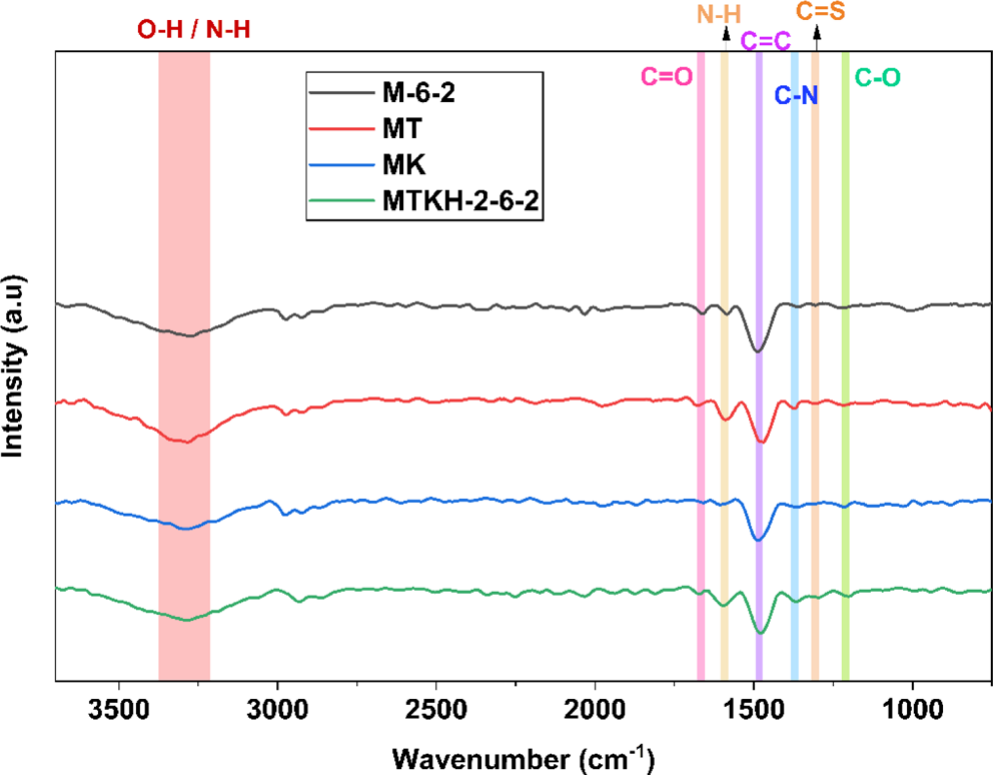
FTIR spectra of pristine and chemically modified biochar.

The band near 1586 cm^–1^ is attributed to N–H bending vibrations, further supporting the presence of amine functionalities in MT and MTKH-2–6–2, which are commonly regarded as active adsorption sites in heteroatom-functionalized carbons. The adsorption peak around 1483 cm^–1^ corresponds to aromatic C=C stretching vibrations, indicating the development of partially ordered carbon frameworks during carbonization and activation. The band at approximately 1281 cm^–1^ is assigned to C–N stretching vibrations and becomes more intense after thiourea treatment, confirming nitrogen incorporation into the carbon matrix. In addition, the weak band near 1318 cm^–1^, corresponding to C=S stretching vibrations, appears only in thiourea-modified samples, indicating the successful introduction of sulfur-containing functionalities. The coexistence of N and S containing groups consistent well with previous reports on heteroatom-doped carbons, where such functionalities were shown to create chemically diverse adsorption environments favorable for CO_2_ capture.^38^ Meanwhile, the band near 1215 cm^–1^, associated with C–O stretching vibrations, remains relatively unchanged across all samples, suggesting that oxygen-containing functionalities mainly originates from the biomass precursor itself. The FTIR results demonstrate that thiourea modification mainly tailors the surface chemistry through the incorporation of N and S containing functionalities, whereas chemical activation predominantly contributes to structural development. The synergistic presence of amine, carbonyl, and heteroatom functionalities in MTKH-2-6-2 provides diverse interaction pathways for CO_2_ adsorption, which collectively contribute to its superior adsorption performance.

The high-resolution N 1s XPS spectra for M-6-2 and MK exhibit two major deconvoluted peaks at approximately 398.5 and 400.3 eV, corresponding to pyridinic-N and pyrrolic-N species, respectively, as shown in Figure 9.^39^ These nitrogen species are commonly associated with edge-defect sites and heterocyclic structures within the carbon framework. Their presence in both samples suggests that a portion of nitrogen is inherently retained from the biomass precursor and stabilized during high-temperature treatment. Although M-6-2 and MK exhibit similar nitrogen configurations, MK shows a comparatively higher relative intensity of pyridinic-N, suggesting that K_2_C_2_O_4_ activation facilitates the stabilization of edge-type nitrogen functionalities without substantially increasing the overall nitrogen content. This observation is consistent with the CHNSO results, where MK exhibits slightly lower nitrogen content than M-6-2, further indicating that chemical activation predominantly influences pore development rather than nitrogen incorporation.^40^ Such pyridinic and pyrrolic-N species were consistent with the reported studies in biomass-derived carbons and are recognized as important active sites for CO_2_ adsorption because they provide localized electron-rich regions that can strengthen interactions with CO_2_ molecules.^41,42^

**Figure 9:**
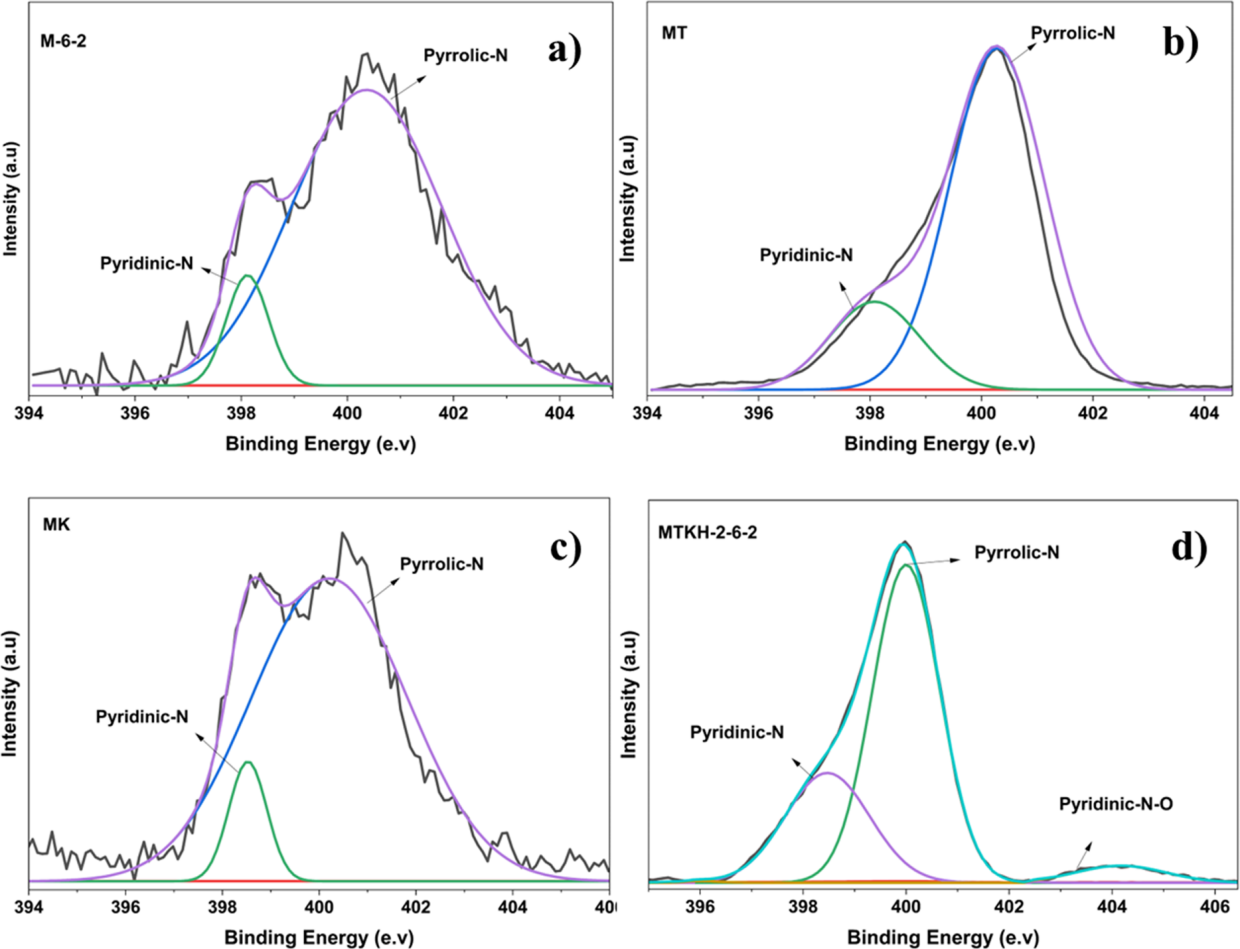
High-resolution XPS N 1s spectra of (a) M-6-2, (b) MT, (c) MK, and (d) MTKH-2-6-2.

In contrast, the thiourea-modified samples MT and MTKH-2-6-2 exhibit similar pyridinic-N and pyrrolic-N peaks with noticeably higher intensities, reflecting the increased nitrogen incorporation confirmed by CHNSO analysis. In addition, MTKH-2-6-2 shows an additional peak near 403.8 eV, attributed to oxidized pyridinic-N species. The appearance of this peak suggests partial oxidation of nitrogen functionalities during the combined thiourea modification and K_2_C_2_O_4_ activation process. Such oxidized nitrogen species have been associated with increased surface polarity and improved CO_2_ affinity through dipole–quadrupole and acid–base interactions. This observation agrees well with the FTIR results, which revealed enhanced N–H and C–N vibrations in the thiourea-modified samples, together with the higher nitrogen contents observed from CHNSO analysis. Finally, the XPS results indicate that thiourea treatment not only increases the nitrogen content but also diversifies the nitrogen bonding environments within the carbon matrix, whereas K_2_C_2_O_4_ activation primarily contributes to structural development and stabilization of surface nitrogen functionalities. The coexistence of pyridinic-N, pyrrolic-N, and oxidized nitrogen species therefore provides multiple adsorption sites that collectively enhance the interaction between the carbon surface and CO_2_ molecules.

### 3.2 Structural–Property Performance Relationship

The combined characterization results presented in Sections 3.1.1–3.1.3 collectively demonstrate that the CO_2_ adsorption performance of the developed sorbents is governed by the combined influence of pore architecture and surface chemistry, where neither factor independently explains the overall adsorption behavior. The textural characteristics, particularly surface area and microporosity, primarily control the adsorption capacity, whereas heteroatom-containing functionalities and defect sites mainly influence the strength of CO_2_ surface interactions. This relationship is clearly reflected in the comparison between MT and MK. CHNSO, FTIR, and XPS analyses confirmed that MT possessed comparatively higher N and S incorporation together with more abundant surface functionalization than MK. Nevertheless, BET analysis showed that MK exhibited substantially higher surface area and micropore volume, resulting in superior CO_2_ adsorption performance despite its lower heteroatom content. These findings indicate that although surface functionalities enhance adsorption affinity, well-developed microporosity remains the dominant factor governing overall CO_2_ uptake, while heteroatom-containing groups primarily act as complementary adsorption-promoting sites.

Among all investigated sorbents, MTKH-2-6-2 was the only sample in which both pore characteristics and surface chemistry were simultaneously optimized. BET and NLDFT analyses confirmed extensive micropore development together with enhanced surface area, while SEM observations revealed a highly accessible interconnected porous network favorable for CO_2_ diffusion. Raman analysis further indicated increased structural disorder and defect density, which can generate additional active adsorption sites, whereas CHNSO, FTIR, and XPS analyses confirmed the successful incorporation of N and S containing functionalities within the carbon framework. Collectively, these characterization results demonstrate that the superior adsorption performance of MTKH-2-6-2 arises from the synergistic interplay between optimized microporosity, enriched surface chemistry, and increased defect density rather than from any single parameter independently, thereby establishing a clear structure–property–performance relationship within the developed sorbent system.^43,44^

### 3.3 Two-Level Optimization

The two-level optimization strategy focuses on optimizing both adsorbent synthesis and CO_2_ adsorption conditions. In Level I screening, adsorbent-related parameters including the selection of raw materials, pyrolysis conditions, synthesis protocols, precursor weight ratios, and activation conditions were optimized to improve the structural and surface properties of the adsorbent, which are critical for efficient CO_2_ capture. In Level II, the CO_2_ adsorption process was standardized and optimized using RSM to identify the ideal operating conditions for maximizing CO_2_ uptake capacity. This was followed by model validation to ensure the reliability, reproducibility, and overall performance consistency of the developed adsorbent system.

#### 3.3.1 Hierarchical Optimization of Adsorbent (Level I)

##### 3.3.1.1 Selection of Biomass

The selection of an appropriate precursor plays a crucial role in the development of effective biochar-based adsorbents. Biomass mainly consists of three major lignocellulosic components: cellulose, hemicellulose, and lignin, each contributing differently to biochar formation and pore evolution during pyrolysis.^45^ Cellulose and hemicellulose tend to create microporous frameworks, whereas lignin owing to its higher thermal stability, contributes to the structural rigidity and carbon yield. However, biomass with excessively high lignin content often results with lower surface area. Therefore, the selection of biomass with a balanced lignocellulosic composition is important for achieving favorable adsorption characteristics.^46^ Table S1 summarizes the lignocellulosic composition of commonly reported biomass precursors employed for CO_2_ capture applications. Among the different biomass sources, deoiled cakes are particularly attractive because of their relatively balanced distribution of cellulose, hemicellulose, and lignin, although their application in CO_2_ adsorption remains comparatively underexplored. The detailed lignocellulosic composition of the deoiled cake biomasses investigated in this study, as reported in the literature, is presented in Table S2.

In addition to their balanced lignocellulosic composition, deoiled cakes also contain a considerable amount of protein, which is advantageous to capture CO_2_. During pyrolysis, these proteins facilitate the in situ incorporation of nitrogen-containing functionalities onto the carbon surface, thereby improving surface basicity and enhancing affinity toward acidic CO_2_ molecules.^47^ Furthermore, the naturally occurring alkali and alkaline earth metals present within the biomass can act as self-activating agents during carbonization, thereby contributing to pore development and enhanced adsorption properties.^48^

Table S3 summarizes the adsorption performance of the selected biomass precursors together with their elemental composition obtained from EDX analysis. Among the investigated materials, mahua and mustard deoiled cakes exhibited the highest CO_2_ adsorption capacities. Their improved performance can be attributed to the combined influence of balanced lignocellulosic composition, relatively higher protein content, and the presence of mineral species such as Na, P, Mg, K, and Ca. N-containing functionalities derived from proteins can enhance interactions with CO_2_ molecules through acid–base and dipole–quadrupole interactions.^49^ In parallel, oxygen-containing surface groups including hydroxyl, carbonyl, ether, carboxyl, and phenolic functionalities originating from cellulose, hemicellulose, and lignin also contribute to CO_2_ adsorption.^50^ The coexistence of heteroatom functionalities and inorganic mineral constituents can further promote CO_2_ capture through the combined contribution of physisorption and weak chemisorption interactions involving acid–base and electrostatic interactions.^51^ The screening results suggest that biomass precursors possessing balanced lignocellulosic composition, sufficient protein content, and naturally occurring mineral constituents are highly favorable for producing biochar’s with enhanced surface chemistry and adsorption characteristics, thereby highlighting deoiled cakes as promising precursors for carbon capture.

##### 3.3.1.2 Effect of Textural Properties on the CO_2_ Adsorption

The textural characteristics play a significant role in governing CO_2_ adsorption performance, as discussed in Section 3.1.1. Table S4 illustrates the notable differences in specific surface area, total pore volume, and pore size distribution among the optimized sorbents, which directly influence their CO_2_ capture capacity. A clear distinction was observed between Ma-5-2 (Mahua-500 °C-2 h) and M-5-2 (Mustard-500 °C-2 h). Despite being synthesized under similar carbonization conditions, M-5-2 exhibited a comparatively higher surface area and pore volume than Ma-5-2, resulting in a slightly higher CO_2_ uptake of 0.61 mmol/g compared with 0.46 mmol/g for Ma-5–2, which highlights the importance of surface area together with micromesoporous characteristics in enhancing CO_2_ adsorption. The superior performance of M-5-2 is primarily attributed to the favorable biochemical composition of mustard biomass, which possesses an optimal ratio of cellulose, hemicellulose, and lignin along with a comparatively higher protein content than mahua biomass. During carbonization, cellulose and hemicellulose undergo thermal decomposition, releasing volatile compounds that promote pore formation, whereas lignin undergoes progressive aromatization and condensation, contributing to a rigid and thermally stable carbon framework.^52^ Furthermore, the carbonization temperature and residence time significantly influence the textural properties. Increasing the temperature (500–600 °C) and residence time (1.5–2 h) results in a substantial enhancement in specific surface area and micropore development due to improved devolatilization and intensified aromatic condensation reactions, which facilitate micropore formation.^53^ Such microporous structures are highly favorable for CO_2_ adsorption. However, further increases in temperature (600–700 °C) and time (2–2.5 h) resulted in a decline in surface area and pore volume, mainly due to pore widening and partial structural collapse under severe carbonization conditions. A similar trend was reported by Chatterjee et al.,^54^ in biochar’s derived from miscanthus, switchgrass, and corn stover, where increasing the carbonization temperature from 700 to 800 °C caused a significant reduction in both surface area and pore volume, leading to a decrease in CO_2_ uptake from 2.8 to 1.7 mmol/g. Likewise, Ringsby et al.,^55^ observed that variations in carbonization temperature and residence time induced considerable differences in the textural characteristics of the derived carbon materials. Comparable findings were also reported by Akter et al.,^56^ who observed that increasing the carbonization temperature from 600 to 650 °C reduced the surface area from 1352 to 675 m^2^/g, accompanied by a decrease in CO_2_ adsorption capacity from 3.25 to 2.75 mmol/g. These results collectively emphasize the importance of optimizing carbonization parameters to achieve desirable textural properties for effective CO_2_ capture.

In addition to carbonization parameters, the synthesis route plays a critical role in bringing chemical modifiers into contact with carbon distinctly, resulting in significant variations in textural properties. Among the investigated approaches, the hybrid synthesis pathway exhibits the highest CO_2_ adsorption capacity along with a substantial specific surface area and total pore volume, confirming the development of a highly accessible and well-interconnected porous network.^57^

A clear distinction was observed between the impregnation, solvent-free, and hybrid synthesis routes. The impregnation method enables solvent-assisted diffusion of activating agents into the carbon matrix, resulting in more effective pore development compared to the physical dry mixing involved in the solvent-free approach. This enhanced interaction between the activating agent and carbon framework promotes improved surface area and pore formation. A comparable observation was reported by Manyà et al.^58^ for vine-shoot-derived biochar activated using KOH, where the impregnation method produced a substantially higher surface area (1672 m^2^/g) than the solvent-free approach (1032 m^2^/g), consequently increasing the CO_2_ adsorption capacity from 1.92 to 2.42 mmol/g. The hybrid-derived sample exhibited the highest specific surface area along with well-developed micromesoporous characteristics, which contributed to its superior adsorption efficiency. The enhanced microporosity provides abundant active adsorption sites for CO_2_ capture, while the mesoporous channels improve pore accessibility and facilitate efficient mass transfer. Sevilla et al.^59^ also demonstrated that a well-developed micromesoporous architecture significantly enhances CO_2_ adsorption performance by improving both adsorption capacity and diffusion efficiency. Therefore, the superior adsorption performance of the hybrid-derived adsorbent can be attributed to the combined contribution of higher surface area, well-developed micromesoporous structure, and improved pore connectivity.^54^

Nandi et al.^60^ demonstrated that the optimization of activating agent (KOH) ratio is crucial, as inadequate loading limits pore development, whereas excessive loading reduces surface area due to severe etching effects. Similarly, Varghese et al.^11^ optimized the melamine and K_2_CO_3_ ratio and observed a substantial increase in surface area from 714 to 1528 m^2^/g with increasing K_2_CO_3_ loading, followed by a decline in CO_2_ uptake at higher K_2_CO_3_ ratios due to reduced surface area. In addition, optimization of melamine content resulted in only a slight increase in surface area, whereas excessive melamine loading caused a significant reduction because of pore blockage. Collectively, these studies highlight that careful optimization of activating agents and nitrogen precursors is essential for enhancing surface area, maintaining pore accessibility, and achieving superior CO_2_ adsorption performance. A comparable trend was observed in the present study, where the weight ratio between the precursor, activating agent, and nitrogen source played a critical role in governing pore evolution and structural stability. Variations in K_2_C_2_O_4_ loading strongly influenced the development of surface area and pore volume. In the absence of an adequate activating agent, as observed for MTKH-1, pore generation is limited, resulting in a low surface area, whereas excessive K_2_C_2_O_4_ leads to overetching, pore widening, and partial structural collapse, thereby reducing the accessible surface area in MTKH-3. These observations indicate that both inadequate and excessive activating agent are detrimental to pore development. Meanwhile, MTKH-4 and MTKH-5 exhibited only marginal differences in textural characteristics, emphasizing the importance of optimizing the nitrogen precursor ratio.^61^ Thiourea primarily contributes to surface chemical modification rather than direct pore generation. Therefore, insufficient thiourea loading limits effective heteroatom incorporation, whereas excessive loading can induce partial pore blockage, thereby reducing accessible porosity. Among the investigated samples, MTKH-2 achieved an optimal balance between thiourea and potassium oxalate, resulting in superior textural properties compared to the other compositions. In addition to precursor ratios, activation temperature and residence time also exert a strong influence on pore architecture and surface chemistry, thereby directly affecting CO_2_ adsorption performance.^62^ Analogous to the carbonization trends, increasing the activation temperature from 500 to 600 °C and extending the activation time from 1.5 to 2 h results in a significant increase in specific surface area and total pore volume, accompanied by notable micropore development. These enhancements in textural characteristics lead to a higher CO_2_ uptake of 1.56 mmol/g, indicating the formation of microporous structures favorable for adsorption and gas–solid interactions. However, further increasing the activation temperature and time to 700 °C and 2.5 h resulted in a decline in CO_2_ uptake despite maintaining a relatively high surface area. This reduction can be attributed to pore widening, partial collapse of microporous structures, and the increased formation of mesopores, which reduced the effectiveness of adsorption-active micropores. These results are consistent with reported studies. Chen et al.^44,63^ observed maximum CO_2_ uptake of 5 mmol/g at 298 K for urea and KOH modified biochar activated at 650 °C, while both lower and higher activation temperatures resulted in reduced surface area and inferior adsorption performance.

Collectively, these results indicates that the synthesis pathway and activation parameters critically govern the textural properties of the developed adsorbents, which subsequently determine their CO_2_ adsorption performance. Both inadequate and excessively harsh activation conditions adversely affect pore development. Insufficient activation limits pore generation and surface area development, whereas excessive activation can induce pore widening, and partial structural degradation, thereby reducing the availability of adsorption-active micropores. In contrast, controlled pyrolysis and activation at 600 °C for 2 h promoted favorable micropore development together with enhanced surface area, leading to improved CO_2_ adsorption efficiency. Furthermore, the formation of a well-developed micromesoporous structure through hybrid synthesis and optimized precursor ratios enhanced pore accessibility and facilitated efficient CO_2_ diffusion within the carbon matrix. These observations demonstrate that optimized pore architecture and balanced pore size distribution play a crucial role in achieving superior CO_2_ adsorption performance.

##### 3.3.1.3 Effect of Elemental Composition on the CO_2_ Adsorption

In addition to textural properties, the elemental composition of carbon materials also plays a crucial role in governing CO_2_ adsorption behavior. Surface heteroatoms and naturally occurring inorganic species in biochar can significantly influence the interaction between CO_2_ molecules and the adsorbent surface through dipole–quadrupole interactions, Lewis acid–base interactions, and hydrogen bonding.^64^ Table S4 presents the complete CHNSO elemental composition of all optimized sorbents. A clear distinction was observed between the initially screened Ma-5-2 and M-5-2 sorbents, mainly due to differences in their inherent protein and lignocellulosic compositions. The M-5-2 precursor contained comparatively higher protein and lignocellulosic content than Ma-5-2, which promoted greater heteroatom retention after carbonization.^65^ Consequently, the CO_2_ adsorption capacity increased from 0.46 mmol/g for Ma-5-2 to 0.61 mmol/g for M-5-2, highlighting the influence of precursor composition on adsorption behavior.

Further optimization of carbonization temperature and residence time resulted in systematic changes in elemental composition. Increasing the carbonization temperature from 500 to 600 °C increased the C content due to enhanced devolatilization and aromatization, while the H, N, S, and O contents gradually decreased because of dehydration, decarboxylation, and deamination reactions.^66^ However, at 700 °C, a reduction in C content was observed because of excessive carbon burnoff during activation. A similar trend was observed with increasing residence time, where C enrichment improved up to 1.5–2 h, whereas prolonged treatment for 2.5 h resulted in partial carbon loss due to overactivation. Correspondingly, the H/C and O/C atomic ratios decreased with increasing temperature and residence time, indicating enhanced aromaticity, thermal stability, and hydrophobicity of the developed sorbents.^67^ Similar observations were reported by Li et al.^68^ and Xu et al.,^69^ that excessive thermal severity reduces surface heteroatom content and adversely affects adsorption performance.

Following the optimization of pyrolysis conditions, the influence of different synthesis routes on the elemental composition and adsorption behavior of the developed sorbents was further investigated. A clear distinction was observed between the impregnation and solvent-free synthesis routes. The solvent-free approach exhibited comparatively higher N and S retention, as heteroatom incorporation mainly occurred at defect sites, edge carbons, and near-surface aromatic structures, where chemical bonding is thermodynamically favorable. In addition, the absence of solvent dilution promoted localized precursor decomposition, resulting in improved surface doping and heteroatom retention.^29^ In contrast, the impregnation route resulted in comparatively lower N and S contents because part of the heteroatom-containing species decomposed into volatile compounds such as NH_3_, SO_2_, and H_2_S during thermal treatment, leading to volatilization losses from the carbon matrix.^23^ Nevertheless, the impregnation method generated comparatively higher surface area and pore volume owing to the activating effect of K_2_C_2_O_4_.

The weight ratio of activating and doping agents is another important parameter influencing the elemental composition and adsorption performance of the prepared sorbents. Among MTKH-1, MTKH-2, and MTKH-3, no major differences in N and S contents were observed, indicating that variations in K_2_C_2_O_4_ loading had only a limited influence on heteroatom incorporation. Instead, the changes mainly affected the textural properties through differences in the extent of chemical activation.^32^ In contrast, noticeable differences were observed for MTKH-4 and MTKH-5 due to changes in thiourea loading. MTKH-4 exhibited lower N and S contents because of insufficient thiourea availability, whereas excessive thiourea loading in MTKH-5 likely promoted the formation and clustering of less effective N and S containing surface species. This reduced the accessibility of active adsorption sites and weakened CO_2_ surface interactions, ultimately lowering the adsorption capacity.^70^ These findings indicate that both insufficient and excessive heteroatom precursor loading adversely affect adsorption performance.

Increasing the activation temperature (500–700 °C) and duration (1.5–2.5 h) progressively reduced the N and S contents because of thermally induced deamination and desulfurization reactions, which correlated with lower CO_2_ uptake at higher thermal severity.^71^ However, the primary influence of activation conditions was associated with pore development rather than heteroatom incorporation. Activation using K_2_C_2_O_4_ effectively etched the carbon framework and promoted the formation of micro and mesoporosity, whereas thiourea mainly contributed to surface functionalization and heteroatom incorporation. Therefore, the synthesis route predominantly governed the elemental composition and surface chemistry of the adsorbents, while the activation parameters mainly controlled pore evolution and structural development. The results demonstrate that precursor selection, pyrolysis conditions, synthesis pathway, precursor ratio, and activation parameters collectively influence the adsorption behavior of the developed sorbents. Nevertheless, surface chemistry alone was insufficient to achieve superior CO_2_ adsorption performance. Instead, enhanced adsorption efficiency was achieved through a balanced combination of suitable heteroatom incorporation together with well-developed and accessible pore architecture. Overall, the synthesis strategy and activation conditions emerged as the most influential parameters because they collectively determined both effective surface modification and favorable pore development within the carbon framework.

#### 3.3.2 Statistical Optimization of CO_2_ Capture Variables (Level-II)

##### 3.3.2.1 Process Optimizations Using RSM

In this study, RSM was employed to establish a systematic experimental framework for evaluating the carbon capture process. The experiments were conducted at three coded levels (−1, 0, +1) using four key process parameters, namely temperature, gas flow rate, adsorbent dosage, and residence time, as presented in Table 3. These parameters were selected as the independent variables for optimization, while the feed gas composition, inlet pressure (1 atm), quartz column dimensions, and adsorbent material were maintained constant throughout all experimental runs. This design was adopted to investigate the individual and interactive effects of multiple operating conditions on CO_2_ capture performance. The CCD approach was selected because it effectively evaluates process behavior over a broad experimental range while accounting for curvature in the response through quadratic model development. In addition, the inclusion of center and axial points improves model accuracy and facilitates reliable prediction of the optimum operating conditions, thereby enhancing the robustness of the optimization study.

**Table 3:** Parameters/Numeric Factors

variables	–1	0	+1
temperature (°C)	30	45	60
flow rate (mL/min)	100	150	200
loading amount (g)	3	5.5	8
time (min)	60	120	180

The CO_2_ capture results obtained under the CCD-generated experimental conditions are presented in Table S5. The results clearly indicate that increasing the adsorption temperature progressively decreased the CO_2_ capture capacity. The adsorption capacity decreased from 4.9 mmol/g at 30 °C to 3.24 mmol/g at 60 °C. This trend is consistent with previous reports on various modified biochar’s, which generally exhibit higher CO_2_ uptake at lower temperatures and reduced adsorption capacities at elevated temperatures. For instance, Zhang et al.^18^ reported that KOH activated pine-derived biochar exhibited a CO_2_ adsorption capacity of 6.15 mmol/g at 0 °C, which decreased to 3.79 mmol/g at 25 °C. This reduction is mainly attributed to the exothermic nature of CO_2_ adsorption on biochar, where increasing temperature weakens the adsorptive interactions and promotes desorption. Similarly, Wu et al.^57^ developed a K_2_CO_3_ activated, urea-doped biochar that demonstrated adsorption capacities of 7.52 mmol/g at 0 °C and 3.60 mmol/g at 25 °C under 1 bar. Adsorption temperature is therefore a critical parameter influencing CO_2_ capture performance. As shown in Figure 10a, the adsorbent exhibited the highest CO_2_ uptake at lower temperatures, followed by a gradual decline with increasing temperature. This behavior can be attributed to the increased kinetic energy of CO_2_ molecules at elevated temperatures, which enhances molecular motion and weakens the interactions between the gas molecules and the adsorbent surface, ultimately resulting in lower adsorption capacity.

**Figure 10:**
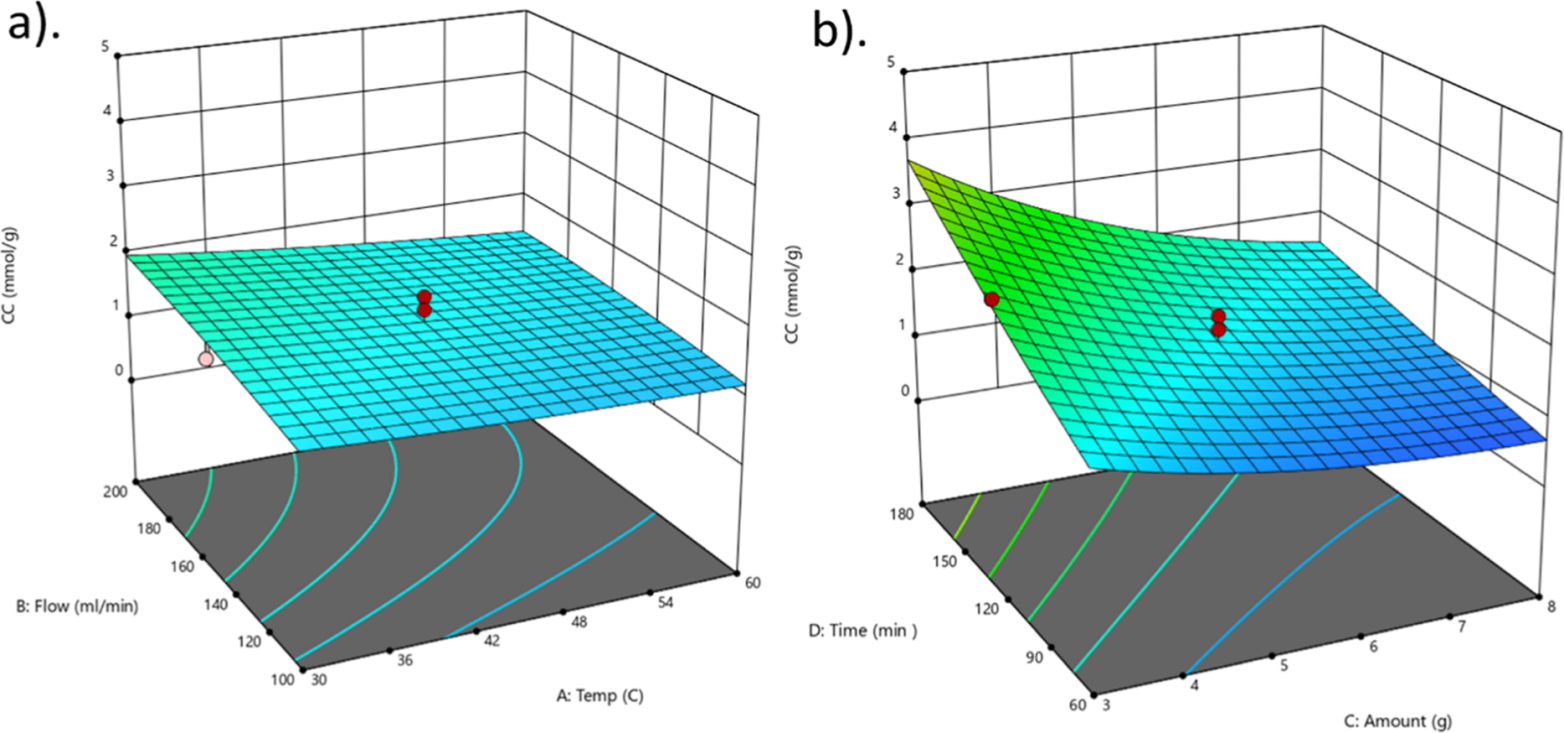
Adsorption capacity with respect to (a) temperature vs flow rate and (b) amount vs time.

The process flow rate is another key parameter that significantly influences the gas–solid interaction in the fixed-bed reactor. In general, excessively high gas flow rates shorten the residence time of CO_2_ molecules inside the adsorption bed, resulting in premature breakthrough and reduced adsorption efficiency due to insufficient contact with the active adsorption sites. In contrast, very low flow rates may prolong the residence time but simultaneously decrease gas throughput and limit the overall adsorption rate.^72^ In the present study, the flow rate was varied between 100 and 200 mL/min based on the operating constraints of the MFC system. Within this investigated range, the CO_2_ adsorption capacity increased from 3.9 mmol/g at 100 mL/min to 4.9 mmol/g at 200 mL/min, as illustrated in Figure 10a. The observed enhancement in adsorption performance at higher flow rates can be attributed to improved external mass-transfer characteristics and increased availability of CO_2_ molecules at the adsorbent surface. Nevertheless, the observed trend in the present study should be interpreted within the investigated operating window, as the highest flow-rate condition was simultaneously associated with the lowest adsorbent loading (3 g) and the longest carbonation duration (180 min), which together ensured sufficient gas–solid interaction prior to breakthrough. Therefore, the improved adsorption performance observed at 200 mL/min should not be generalized to substantially higher flow conditions. Since the experimental flow range was restricted by the operational limit of the MFC system, the actual optimum flow rate could not be definitively established. At considerably higher gas velocities, reduced residence time and accelerated breakthrough behavior are expected to adversely affect the overall CO_2_ capture efficiency.

Figure 10b illustrates the influence of adsorbent amount on CO_2_ uptake. The adsorption capacity decreased from 4.9 mmol/g to 1.92 mmol/g when the adsorbent loading increased from 3 to 8 g. A similar trend was reported by Ghaemi and Behroozi,^73^ where increased sorbent loading reduced the effective gas–solid interaction due to particle accumulation and restricted accessibility of active sites. Sreenivasulu et al.^74^ also observed that excessive adsorbent loading can increase bed resistance and contribute to pressure drop within the reactor, thereby reducing the overall adsorption performance. In the present study, higher adsorbent loading likely caused increased diffusion resistance and restricted gas distribution within the packed bed, limiting the efficient transport of CO_2_ molecules to the available adsorption sites. Consequently, lower adsorbent loading provided better utilization of active sites and improved adsorption performance under the investigated conditions.

Carbonation time also showed a significant influence on the adsorption performance. The CO_2_ uptake increased with increasing carbonation time, indicating that prolonged exposure allowed greater interaction between CO_2_ molecules and the adsorbent surface. Longer contact duration provides sufficient time for diffusion and occupation of available active sites before saturation occurs. Li and co-workers^75^ observed the similar trend, where extended carbonation periods improved adsorption efficiency due to enhanced gas–solid interaction and progressive surface coverage of CO_2_ molecules. However, once the adsorption system gradually approaches equilibrium, further increases in carbonation time may result only in marginal improvement in adsorption capacity.

##### 3.3.2.2 Model Validation

The relevant optimal experimental conditions are summarized in Table 4. The developed model equations establish a quantitative relationship between CO_2_ adsorption capacity and the selected process variables, namely temperature, gas flow rate, adsorbent amount, and residence time. In the coded model equations, A, B, C, and D represent temperature, flow rate, adsorbent amount, and residence time, respectively. Both coded and actual factor equations are provided in the Supporting Information to ensure clarity and reproducibility of the modeling methodology.

**Table 4:** Optimum CC Conditions Obtained from the RSM Model Verification

adsorbent	temperature (°C)	flow rate (mL/min)	amount (g)	time (min)	predicted CC (mmol/g)	experimental CC (mmol/g)
MTKH-2-6-2	30	200	3	180	4.6	4.9

The robustness and adequacy of the developed RSM model were assessed through ANOVA analysis. The quadratic model exhibited high statistical significance, with a model *F*-value of 29.86 and a corresponding *p*-value of <0.0001, confirming that the developed model adequately represented the experimental data within the investigated operating range. Furthermore, the nonsignificant lack-of-fit value (*F* = 1.64, *p* = 0.3058) demonstrated satisfactory agreement between the predicted and experimental responses. The high coefficient of determination *R* ^2^ (0.96), along with the adjusted *R* ^2^ (0.93) and predicted *R* ^2^ (0.81), further verified the good predictive capability of the model. In addition, the low standard deviation (0.0918) and coefficient of variation (7.29%) reflected good experimental precision and reproducibility.

Among the investigated parameters, adsorbent amount (*C*) and carbonation time (*D*) exerted the most pronounced influence on CO_2_ adsorption performance, with *F*-values of 160.56 and 200.65, respectively. The strong contribution of carbonation time highlights the importance of sufficient gas–solid contact duration for effective diffusion and adsorption of CO_2_ molecules before breakthrough occurs. Similarly, the significant influence of adsorbent amount suggests that bed configuration and packing density strongly govern gas distribution and accessibility of active adsorption sites within the fixed-bed column. Experimentally, lower adsorbent loading resulted in higher adsorption capacity, which may be attributed to reduced diffusion resistance and improved utilization of accessible surface-active sites. In contrast, excessive bed loading likely promoted localized mass-transfer limitations and restricted gas diffusion through the packed bed.

Temperature (A) and flow rate (B) also significantly affected the adsorption process, although their influence was comparatively lower than that of adsorbent amount and carbonation time. Within the investigated operating range, increasing flow rate enhanced CO_2_ uptake, which can be associated with improved external mass-transfer characteristics and greater CO_2_ availability at the adsorbent surface. However, excessively high gas velocities are generally associated with reduced residence time and accelerated breakthrough behavior, which may adversely affect adsorption performance beyond the investigated range. The significant interaction effect between adsorbent amount and carbonation time (CD) further indicates that prolonged carbonation time becomes more effective at lower bed loading, where CO_2_ molecules can access the available adsorption sites more efficiently. Furthermore, the significant quadratic effect of adsorbent amount (*C* ^2^) confirms the existence of a nonlinear relationship between bed loading and adsorption performance, resulting from the competing influence of increased adsorption site availability and enhanced diffusion resistance at higher packing density. The developed RSM model effectively captured the combined influence of the operating parameters and provided valuable insight into the factors governing CO_2_ adsorption behavior within the present fixed-bed adsorption system.

## 4 Thermo-Kinetic Modeling and Analysis

### 4.1 Adsorption Kinetics

To achieve a more comprehensive understanding of the adsorption mechanism, it is essential to examine the kinetic regimes governing the process. Kinetic modeling plays a pivotal role in elucidating the interactions between the adsorbate and the adsorbent, helping to determine whether the process is dominated by physical adsorption, chemical adsorption, or a combination of both. In this study, kinetic experiments were conducted under the optimized conditions obtained from RSM until equilibrium was attained. The experimental data were analyzed using four kinetic models, namely the pseudo-first-order (PFO), pseudo-second-order (PSO), Elovich, and Weber-Morris models, which are expressed mathematically as follows in eqs 1–4.

1 $$\log\left({q_{e}-q}_{t}\right)=\log\left(q_{e}\right)-\frac{k_{1}}{2.303}t$$

2 $$\frac{t}{q_{t}}=\frac{1}{k_{2}{\left(q_{e}\right)}^{2}}+\frac{1}{q_{e}}t$$

3 $$q_{t}=\frac{1}{\beta }\left(\ln\left(\alpha \cdot \beta \right)+\ln\left(t\right)\right)$$

4 $$q_{e}=k_{d}\sqrt{t}+C$$

Here, the *q*_e_ and *q*_*t*_ denote the amount of CO_2_ adsorbed at equilibrium and CO_2_ adsorbed per unit mass at a specific time *t* in mmol/g. The *k*_1_ and *k*_2_ are associated with the pseudo-first order and pseudo-second-order constants, respectively. *k*_d_ represents the Weber–Morris coefficient for interparticle diffusion. α and β signify the initial adsorption and desorption rates, in Elovich’s model. Equations 1–4 were used to graphically determine the parameters of the corresponding models from Figure 11a–d.

**Figure 11:**
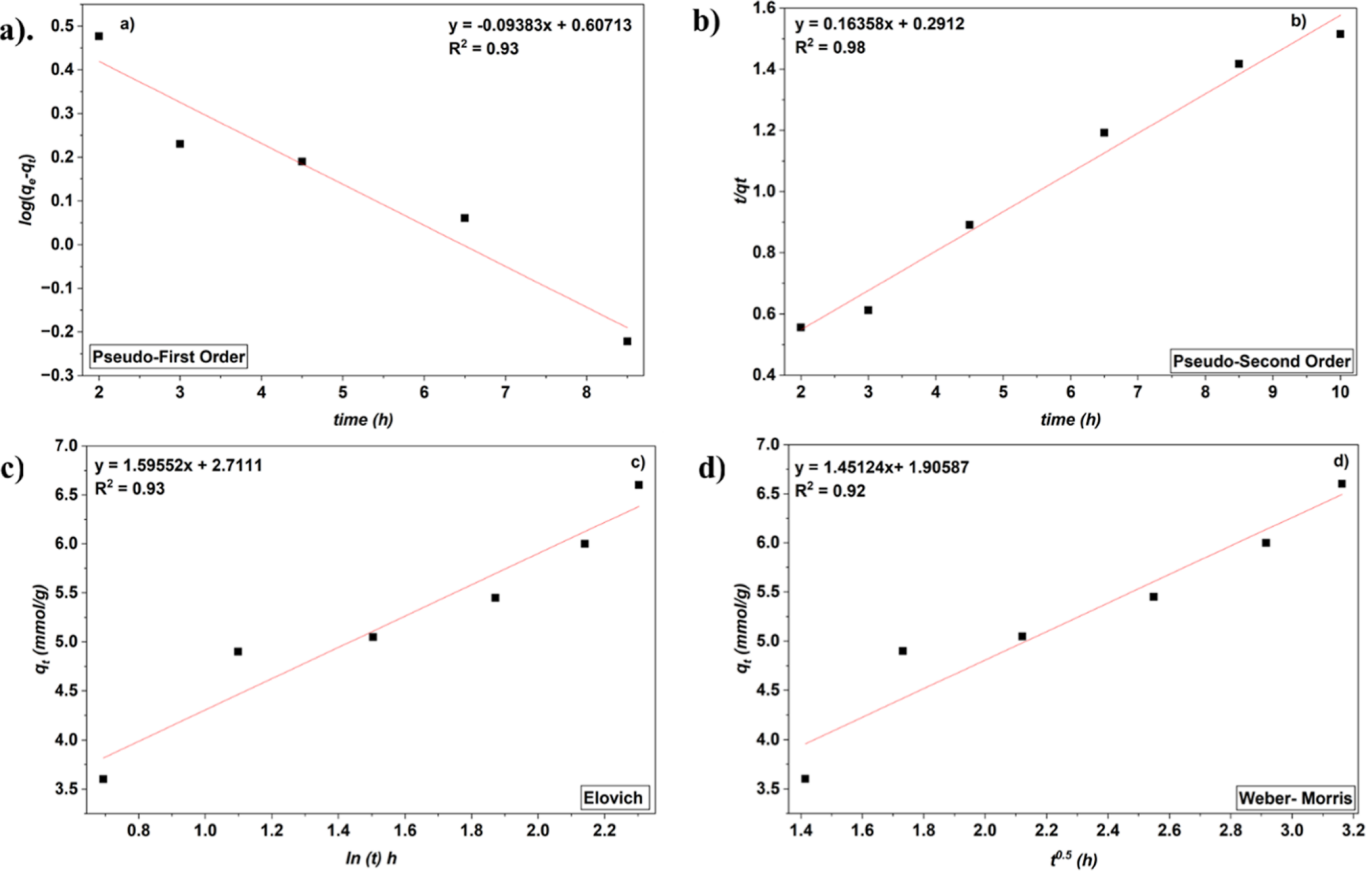
Kinetic modeling fits of (a) Pseudo-first order; (b) pseudo-second order; (c) Elovich model, and (d) Weber-Morris model.

The kinetic parameters obtained from various models for CO_2_ adsorption on MTKH-2-6-2 are summarized in Table S8. Among the evaluated models, the PSO model provided the best Fitt with highest correlation coefficient *R* ^2^ (0.98) with the rate constant *k*_2_ (0.092 g mmol^–1^.h^–1^) and a fitted equilibrium adsorption capacity (*q*_e_)_fit_ of 6.11 mmol/g that closely matches the experimental value (*q*_e_)_exp_ of 6.6 mmol/g. The close agreement between the experimental and fitted adsorption capacities indicates that the CO_2_ adsorption process involves the combined contribution of physisorption and surface-mediated interactions associated with the heteroatom-functionalized carbon framework. In contrast, the pseudo-first-order (PFO) model exhibited comparatively lower fitting accuracy with an *R* ^2^ value of 0.93 together with a lower fitted equilibrium adsorption capacity, indicating that the model could not adequately describe the adsorption behavior of MTKH-2-6-2. This suggests that the adsorption process does not follow simple first-order adsorption kinetics. The Elovich model, which is generally associated with chemisorption on heterogeneous surfaces, also showed relatively lower fitting accuracy with *R* ^2^ (0.83), indicating that although surface heterogeneity is present, the model does not fully represent the overall adsorption kinetics. This behavior may arise from variations in the availability and distribution of active adsorption sites during the adsorption process. Meanwhile, the Weber–Morris intraparticle diffusion model exhibited moderate fitting behavior with *R* ^2^ (0.92), suggesting that intraparticle diffusion contributes to the adsorption process but is not the sole rate-controlling step, thereby indicating the involvement of multiple adsorption stages. The superior fit of the PSO model together with the strong agreement between the experimental and calculated adsorption capacities demonstrates that the PSO model most effectively describes the CO_2_ adsorption behavior of MTKH-2-6-2. These findings indicate that the adsorption process is governed by the combined contribution of weak intermolecular interactions and surface chemical interactions associated with the heteroatom-functionalized carbon surface. Comparable kinetic behavior has also been reported by Monteagudo et al.^15^ and Zhang et al.^31^ for chemically modified biochar’s derived from olive pomace and bamboo, respectively, where CO_2_ adsorption was attributed to the combined influence of physisorption and chemisorption interactions. The consistency of these observations further supports the suitability of the PSO model for describing CO_2_ adsorption on chemically modified biochar adsorbents.

### 4.2 Equilibrium and Adsorption Isotherm Studies

The partial pressure of CO_2_ in the feed stream plays a significant role in determining the adsorption capacity of the adsorbent. To evaluate the influence of CO_2_ concentration, the adsorption experiments were performed under the optimal conditions obtained from the RSM study at different partial pressures ranging from 0.12 to 0.92 with an interval of 0.20 (0.12, 0.32, 0.52, 0.72, and 0.92), as shown in Figure 12. The results demonstrated that the adsorption capacity progressively increases with increasing CO_2_ partial pressure, indicating the strong dependence of CO_2_ uptake on feed concentration. Even at the lowest partial pressure, the adsorbent exhibited a CO_2_ uptake of 6.6 mmol/g, indicating efficient utilization of the available adsorption sites. With further increase in CO_2_ partial pressure, the adsorption capacity continued to increase due to the greater availability of CO_2_ molecules and the progressive occupation of adsorption sites. However, the rate of increase gradually diminished at higher partial pressures, which is characteristic of predominantly microporous adsorbents where the adsorption sites progressively approach saturation.

**Figure 12:**
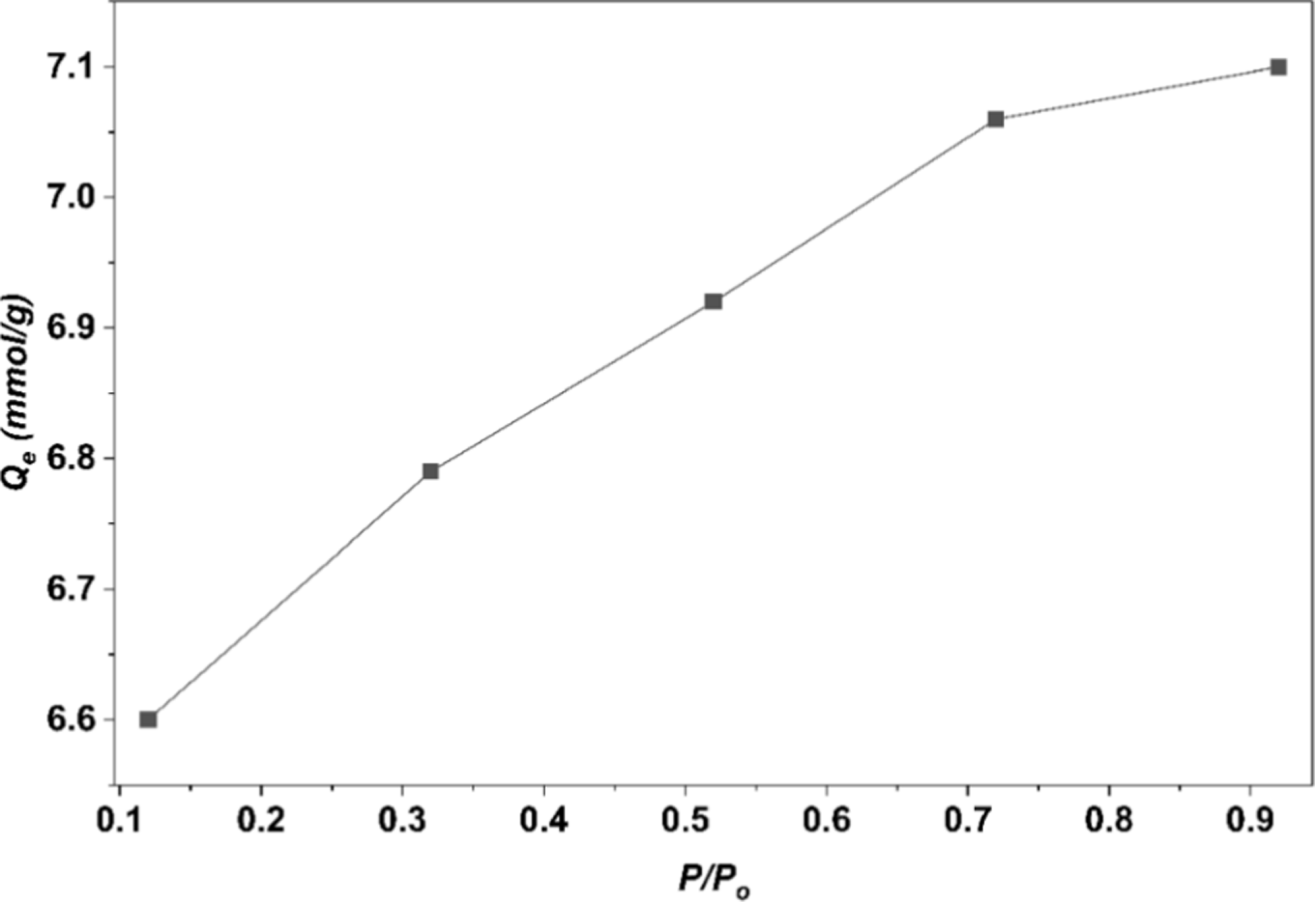
CO_2_ adsorption equilibrium- of MTKH-2-6-2 at 303 K.

The overall adsorption behavior observed for MTKH-2-6-2 resembles a Type I adsorption isotherm, typically associated with microporous materials. Similar trends have been reported by Li et al.^76^ where increasing CO_2_ partial pressure enhanced the adsorption capacity, while the incremental increase gradually decreased at higher pressures due to progressive pore filling. The adsorption profile observed in this study agrees well with the well-developed microporous structure of MTKH-2-6-2 discussed earlier.

Adsorption isotherms are essential for understanding the nature and type of adsorption processes, as they offer critical insights regarding the interactions between the adsorbate and the adsorbent surface at equilibrium conditions. To evaluate the equilibrium adsorption behavior of CO_2_ on MTKH-2-6-2, the experimental data obtained under the optimized conditions predicted by the RSM model were analyzed using the Langmuir and Freundlich isotherm models after equilibrium was attained. The mathematical expressions for these models are provided in eqs 5 and 6.

5 $$\frac{1}{q_{e}}=\frac{1}{K_{L}q_{m}}\frac{1}{P_{{CO}_{2}}}+\frac{1}{q_{m}}$$

6 $$\log\left(q_{e}\right)=\log\left(K_{F}\right)+\frac{1}{n}\log\left(P_{{CO}_{2}}\right)$$

where *q*_e_ (mmol CO_2_ g^–1^ biochar) represents the CO_2_ adsorption capacity of the adsorbent at equilibrium, *q*_m_ denotes the maximum adsorption capacity, and *K*_L_, and *K*_F_ are the respective adsorption constants for the Langmuir and the Freundlich models. *P*_CO_2__ represents the equilibrium pressure, and n is the heterogeneity factor representing the deviation from linear adsorption behavior.

The Langmuir isotherm model exhibits the best fit with the equilibrium adsorption data, achieving a maximum adsorption capacity of 7.16 mmol/g and a correlation coefficient (*R* ^2^) of 0.99, as illustrated in Figure 13 and Table S9. This finding implies that the adsorption of CO_2_ onto the MTKH-2-6-2 adsorbent occurs through a monolayer adsorption process. Conversely, the Freundlich isotherm, with a Freundlich constant *K*_F_ = 7.19 mmol g^–1^ atm^–1/*n*^ and a heterogeneity factor (*n* = 27.93), suggests a variable adsorption capacity linked to surface heterogeneity. While both models display relatively high *R* ^2^ values, indicating a strong agreement between the experimental results and theoretical predictions, the Langmuir model provides a slightly better representation of the adsorption isotherms, as evidenced by its higher *R* ^2^ value (0.99) compared to the Freundlich model (0.95) and the closely matching *q*_m_ values observed in the experiments.^4,18^

**Figure 13:**
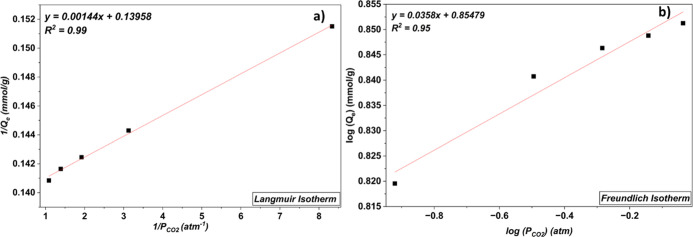
Experimental data fitting with (a) Langmuir and (b) Freundlich isotherm model.

The CO_2_ capture values reported in this study were obtained under different experimental conditions and therefore correspond to different stages of adsorption evaluation. During the initial material screening stage, MTKH-2-6-2 exhibited a CO_2_ uptake of 1.56 mmol/g at 30 °C, 100 mL/min, 3 g adsorbent loading, and 60 min adsorption time, as presented in Table 1. This value was obtained under nonoptimized fixed-bed conditions and was primarily used for comparative assessment of the developed sorbent formulations under identical operating conditions rather than to represent the maximum adsorption performance of the material. Following optimization of the process parameters using RSM, the CO_2_ capture capacity increased to 4.9 mmol/g at 30 °C, 200 mL/min, 3 g adsorbent loading, and 180 min adsorption time, representing the optimized fixed-bed adsorption performance under practically relevant operating conditions. Upon extending the adsorption experiment until equilibrium was attained, the equilibrium adsorption capacity reached 6.6 mmol/g, indicating more complete utilization of the accessible adsorption sites within the adsorbent structure. Furthermore, the Langmuir isotherm model predicted a theoretical maximum adsorption capacity of 7.16 mmol/g, which showed close agreement with the experimentally determined equilibrium value, thereby confirming the suitability of the isotherm model for describing the adsorption system. A similar trend was reported by Ahmadi et al.,^77^ where the CO_2_ adsorption capacity increased from an initial nonoptimized value of 51.41 mg/g to an equilibrium adsorption capacity of 154.90 mg/g under RSM-optimized conditions at 30 °C and 9 bar, while the Freundlich isotherm model exhibited the best fitting behavior with a maximum adsorption capacity of approximately 230.4 mg/g. Similarly, Jedli et al.^78^ reported a CO_2_ adsorption capacity of 5.02 mg/g under RSM-optimized operating conditions, whereas the Langmuir isotherm model predicted a theoretical maximum adsorption capacity of 9.37 mg/g, further demonstrating the expected difference between experimentally obtained dynamic adsorption capacities and model-predicted equilibrium adsorption capacities.

### 4.3 Adsorption Thermodynamics

The heat of adsorption is a crucial factor for assessing the isosteric strength of an adsorbent, determined through the Clausius–Clapeyron equation. In this study, three distinct temperatures of 303, 318, and 333 K were analyzed under three different pressure levels: 1, 2, and 3 bar. A temperature difference of merely 15 K was maintained for accuracy. Figure 14a depicts the relationship between equilibrium adsorption capacity (*q*_e_) and temperature across varying pressures, showing a consistent decline in CO_2_ uptake as temperature rises at all pressures. This trend confirms that the adsorption process is exothermic since increased thermal energy diminishes the interactions between the adsorbate and adsorbent. On the other hand, at a specific temperature, elevating the pressure results in greater adsorption capacity, which signifies a stronger driving force for CO_2_ adsorption.^18^ The trends dependent on both temperature and pressure offer a foundation for estimating the isosteric heat of adsorption and assessing the strength of gas–solid interactions. The average heat of adsorption for MTKH-2-6-2, calculated at an equilibrium adsorption capacity (*q*_e_) of 6.6 mmol/g, was determined to be 40.2 kJ/mol from the linear Clausius–Clapeyron plot of ln *P* vs 1/*T* (Figure 14b). The relatively high value of adsorption enthalpy suggests that the adsorption mechanism involves a combination of physisorption and chemisorption, indicating strong adsorbate–surface interactions, which aligns well with the kinetic findings. Nazir et al.^23^ reported a comparable heat of adsorption value (39.6 kJ/mol) for biochar derived from modified corn starch, further supporting the strong CO_2_ adsorbent interactions found in this investigation.

**Figure 14:**
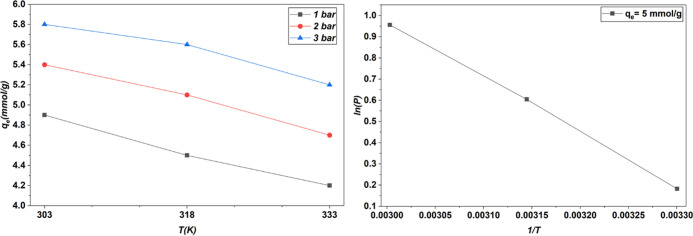
(a). Temperature-dependent adsorption isotherms; and (b). 1/*T* vs ln *P* plot for MTKH-2-6-2.

### 4.4 Adsorption Mechanism

The CO_2_ adsorption mechanism of MTKH-2-6-2 is governed by the synergistic contribution of its hierarchical pore structure and heteroatom-enriched surface chemistry, as illustrated in Figure 15. Among the different adsorption pathways, micropore filling through physisorption plays a significant role in the overall CO_2_ adsorption process. The exceptionally high surface area together with the large micropore volume creates an interconnected network of microporous channels which strongly enhances CO_2_ confinement within the carbon matrix. At such narrow pore dimensions, the close spacing between the pore walls enhances the interaction between the adsorbent surface and CO_2_ molecules, thereby improving CO_2_ confinement and adsorption capacity. Guo et al.^79^ also reported enhanced CO_2_ uptake in walnut shell-derived microporous carbon due to the presence of narrow pore channels and improved microporous confinement. The observed Type I adsorption isotherm together with the excellent Langmuir fitting further supports the predominance of monolayer adsorption within microporous regions.

**Figure 15:**
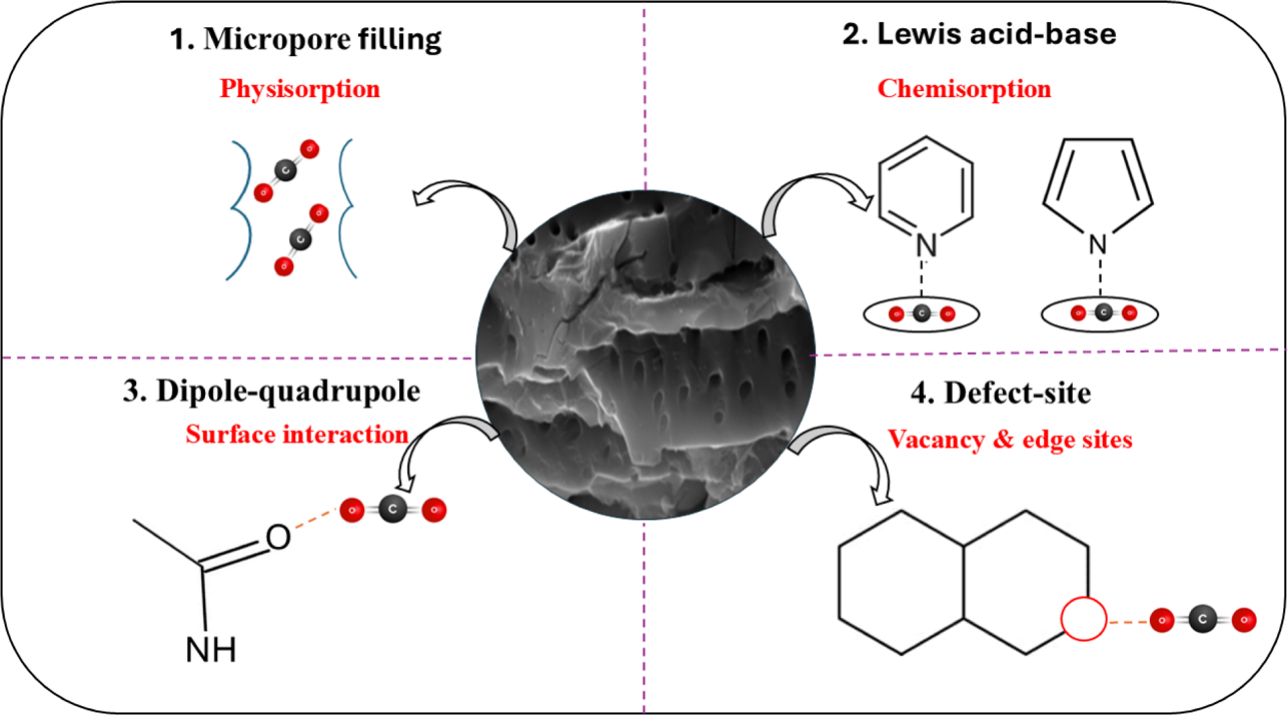
CO_2_ adsorption mechanism of MTKH-2-6-2.

In parallel, chemisorption occurs through Lewis acid–base interactions between the acidic CO_2_ molecules and the heteroatom-containing basic surface sites. Pyridinic-N and pyrrolic-N functionalities, confirmed by XPS N 1s deconvolution peaks at 398.5 and 400.3 eV, respectively, provide localized electron-rich sites that facilitate stronger interaction with CO_2_ molecules. Sun et al.^80^ demonstrated that pyridinic-N and pyrrolic-N functionalities enhance the Bronsted–Lewis basicity of biochar surfaces, thereby promoting the chemisorption of acidic CO_2_ molecules. The FTIR spectra further support the presence of these active functional groups through the presence of characteristic N–H bending, C–N stretching, and C=S vibration bands. In addition, the carbonyl and amide-type groups observed at 1674 cm^–1^ may contribute to dipole–quadrupole interactions with the linear CO_2_ molecule, thereby enhancing the adsorption affinity of the adsorbent surface. A comparable effect was observed by Zhang et al.^18,81^ in pine-derived biochar enriched with oxygen and nitrogen containing surface groups. Furthermore, the higher *I*_D_/*I*_G_ ratio of 0.95 obtained from Raman spectroscopy indicates the presence of a high defect density in MTKH-2-6-2. Such structural defects, including vacancies and edge sites, can act as additional active adsorption centers by enhancing the surface reactivity toward CO_2_ molecules. Previous studies on heteroatom-doped carbon materials have also correlated higher *I*_D_/*I*_G_ values with improved gas adsorption performance.^82^ The calculated isosteric heat of adsorption 40.2 kJ/mol lies within the transitional range between physisorption and chemisorption, where adsorption enthalpy values between 20 and 45 kJ/mol generally indicate the simultaneous contribution of both adsorption mechanisms.^60^ This interpretation is further supported by the excellent fitting of the pseudo-second-order kinetic model, suggesting that surface interactions significantly influence the adsorption rate-controlling step. These collective results indicate that the enhanced CO_2_ adsorption performance of MTKH-2-6-2 arises from the combined contribution of micropore filling, Lewis acid–base chemisorption, dipole–quadrupole interactions, and defect-site adsorption, rather than the dominance of a single adsorption pathway.

## 5 Cyclic Stability Studies

The cyclic stability of MTKH-2-6-2 was evaluated over 20 consecutive adsorption–desorption cycles under the optimized conditions obtained from the RSM study. Throughout the cyclic experiments, the sorbent exhibited excellent stability with only a marginal decline in adsorption performance. The initial CO_2_ uptake of 4.9 mmol/g decreased slightly to 4.6 mmol/g after the 20th cycle, indicating minimal loss in adsorption capacity, as shown in Figure 16. The negligible reduction in performance demonstrates the good regenerability and structural stability of the developed adsorbent during repeated adsorption–desorption operation. The ability of MTKH-2-6-2 to retain high adsorption efficiency under repeated ambient-temperature adsorption and mild regeneration conditions further suggests effective preservation of the accessible adsorption sites and overall structural integrity of the sorbent, highlighting its potential applicability for long-term cyclic CO_2_ capture processes.

**Figure 16:**
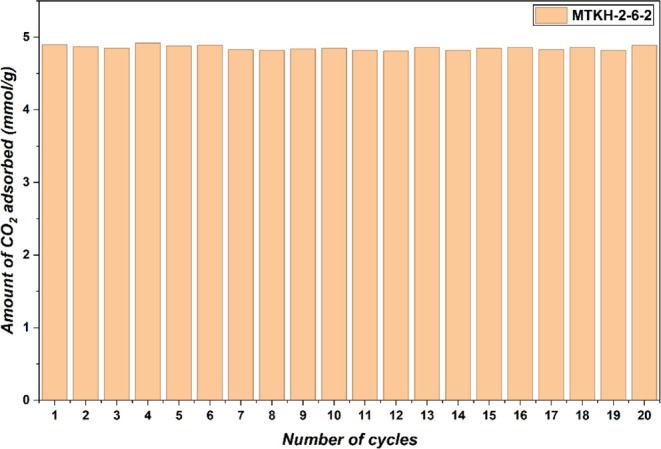
Cyclic stability of MTKH-2-6-2.

## 6 Conclusions

This work investigated the development of a novel functionalized biochar derived from deoiled cake biomass using an integrated multiparameter optimization framework for efficient CO_2_ capture under simulated flue gas conditions. The results demonstrate that the synthesis pathway and activation parameters play a crucial role in determining the physicochemical properties of the adsorbent, which subsequently govern its CO_2_ adsorption performance. Characterization studies revealed a clear structure–property relationship, where the enhanced CO_2_ adsorption performance was attributed to the combined effects of increased microporosity, higher defect density, and the incorporation of N and S containing functional groups, all of which improved adsorption site availability and adsorbate–surface interactions. Process optimization using RSM identified adsorbent amount and contact time as the most influential parameters, with an optimized CO_2_ uptake of 4.9 mmol/g achieved. Kinetic analysis indicates that the adsorption process is governed by a combination of physisorption and chemisorption, suggesting that adsorbate–surface interactions play a key role rather than a single dominant mechanism. The adsorption isotherm behavior followed the Langmuir model, indicating monolayer adsorption, while the average isosteric heat of adsorption, around 40.02 kJ/mol, further supports strong CO_2_ adsorbent interactions. In addition, the adsorbent exhibited excellent cyclic stability with only marginal loss in adsorption capacity over 20 adsorption–desorption cycles. Future studies should focus on evaluating the large-scale applicability of the developed adsorbent, particularly with respect to techno-economic feasibility, regeneration stability over 50–100 adsorption–desorption cycles, and long-term operational performance. Furthermore, a comprehensive evaluation under direct air capture conditions is necessary, as the presence of moisture and competing gaseous impurities may influence adsorption efficiency and regeneration behavior. In addition, comparison with existing commercial CO_2_ sorbents under industrial operating conditions would provide deeper insight into the practical viability, scalability, and potential challenges associated with real-world carbon capture applications.

## Supplementary Material



## Notes

Adsorbent synthesis procedure (Section S1); biomass composition data (Tables S1–S3); physicochemical properties and CO_2_ adsorption performance of screened sorbents (Table S4); RSM experimental results, ANOVA analysis, fit statistics, and coded equations (Tables S5–S7); kinetic and isotherm model parameters (Tables S8 and S9) (PDF)
