# Supplementary material for: Enhanced and Stable Carbon Capture through Hierarchical Sorbent and Process Optimization Using Biochar from Deoiled Cake
Source: ACS Omega. 2026 Jun 29;11(27):40555–74. doi: 10.1021/acsomega.6c03610 (PMC13382662; doi:10.1021/acsomega.6c03610)
Supplement: Supplementary file 1 [file ao6c03610_si_001.pdf]

Supporting Information

Enhanced and Stable Carbon Capture through Hierarchical Sorbent and Process Optimization using Biochar from De-oiled Cake

Sai Krishna Reddy Velagala [a], Arohi Pore [a], Ylias Sabri [b], Rajarathinam Parthasarathy [c], Inkollu Sreedhar [a*]

[a,] Department of Chemical Engineering, BITS Pilani Hyderabad Campus, Hyderabad-50078, India.

[b] Centre of Advanced Materials and Industrial Chemistry, School of Sciences, RMIT University, Melbourne, Victoria 3001, Australia.

[c] Department of Chemical and Environmental Engineering, School of Engineering, RMIT University, Melbourne, Victoria 3001, Australia.

Email: isreedhar@hyderabad.bits-pilani.ac.in

Fax: +91 4066303512; Tel: +91 4066303512

Sec. S1 Synthesis of adsorbents:

S.1.1 Preparation of biochar:

All the de-oiled cakes biomasses were thoroughly washed 3-4 times with water to eliminate dust, residual oil, and pigments. The washed biomasses were sun-dried for 24h, and subsequently oven-dried overnight at 110 °C to remove any residual moisture. The dried samples were then sieved to obtain a uniform particle size range of 150-200 µm. Based on preliminary CO_2 adsorption screening studies (Table S3), mahua (Ma) and mustard (M) were selected for further conversion into biochar at 500 °C (5) for 2h (2), which were named Ma-5-2 and M-5-2.

S.1.2 Synthesis of M-A-B, MTK-C and MTKH-D series:

Following the initial biochar preparation, CO_2 adsorption studies were conducted for both Ma-5-2 and M-5-2. M-5-2 showed superior CO_2 uptake and was therefore selected for further optimization by varying pyrolysis temperature and residence time. Accordingly, Mustard biochar's were prepared at 600 and 700 °C for 2 h (denoted as M-A-B, where A = 6 and 7 correspond to temperatures of 600 and 700 °C, respectively, and B = 2 represents a residence time of 2 h). Based on the screening results (Table S4), the sample produced at 600 °C was selected and further optimized by varying the residence time to 1.5 and 2.5 h (denoted as M-6-B, where B = 1.5 and 2.5 correspond to residence times of 1.5 and 2.5 h, respectively).

The best-performing sample (M-6-2) was subsequently combined with thiourea (T) and potassium oxalate (K) in a 1:1:3 weight ratio and activated at 700 °C for 2 h using different synthesis protocols. The MTK mixtures were prepared via solvent-free (S), impregnation (I), and hybrid (H) methods, denoted as MTK-S, MTK-I, and MTK-H. The hybrid method showed superior performance and was selected for further optimization. Using this method, the MTKH-D series was developed by varying the weight ratios of biochar, thiourea, and potassium oxalate. Following the five compositions (E=1-5), corresponding to ratios of 1:1:2, 1:1:3, 1:1:4,

1:0.5:3, and 1:1.5:3, were synthesized. Among these, MTKH-2 exhibited the best performance and was selected for subsequent activation studies.

S.1.3 Synthesis of MTKH-2-X-Y:

The activation conditions of MTKH-2 were optimized by varying the activation temperature and residence time. MTKH-2 was activated at 500 and 600 ^{o}C for 2h (denoted as MTKH-2-X-Y, where X = 5 and 6 correspond to activation temperatures of 500 and 600 ^{o}C, respectively, and Y = 2 represents a residence time of 2 h). Based on the screening results (Table S4), the sample activated at 600 ^{o}C was selected and further optimized by varying the residence time to 1.5 and 2.5 h (denoted as MTKH-2-6-Y, where Y = 1.5 and 2.5 correspond to residence times of 1.5 and 2.5 h, respectively). The final optimized material (MTKH-2-6-2) was subsequently used for all adsorption studies.

Table S1. Lignocellulosic composition of reported biomass

S. No	Name of the biomass	Cellulose (%)	Hemicellulose (%)	Lignin (%)	Ref
1	Rice husk	32-38	18-19	22-26	[1–3]
2	Wheat straw	35-39	23-36	12-18	[1–3]
3	Bagasse	25-45	25-32	15-25	[1–3]
4	Corn Cob	34-42	32-36	6-16	[1–3]
5	Corn stalk	35-39	17-35	7-19	[1–3]
6	Hardwood	40-55	24-40	18-25	[1–4]
7	Softwood	45-50	25-35	25-35	[1–4]
8	Rice straw	30-35	23-26	17-19	[1–3]
9	Grasses	25-40	25-50	10-30	[1–3,5]
10	Leaves	15-20	80-85	0	[1–3]
11	Agricultural residues	5-15	37-50	25-50	[1–3]

Table S2. Biochemical composition of deoiled cake biomasses

S. No	Name of the biomass	Cellulose (%)	Hemicellulose (%)	Lignin (%)	Protein (%)	Ref
1	Mustard deoiled cake	54-59	25-29	14-16	36	[6–8]
2	Mahua deoiled cake	46-50	25	16	19-20	[9–12]
3	Neem deoiled cake	30-35	16-20	18-20	13-15	[10,13]
4	Castor deoiled cake	10-16	24-26	20-23	31-34	[14,15]
5	Sesame deoiled cake	21-23	33-38	8	30-32	[16,17]
6	Cotton deoiled cake	19-23	19-21	4	24-30	[6,18,19]
7	Coconut deoiled cake	15	28	13	20-25	[20,21]
8	Palm deoiled cake	7.5-12	26-28	15-21	17-18	[22,23]
9	Sunflower deoiled cake	22-26	11-12	9-14	33-36	[24,25]

Table S3. Adsorption performance and elemental composition of selected biomass

S. No	Name of the biomass	CO_2 [a] Uptake (mmol/g)	EDX elemental analysis (wt%)												
			N	S	O	Na	Cl	Mg	K	P	Ca	Si	Cu	Al	Fe
1	Mustard deoiled cake	0.25	11.4	0.17	22.5	0.01	0.01	0.09	0.33	0.16	0.25	0.04	0.14	0.03	0.02
2	Mahua deoiled cake	0.22	6.67	0.09	20.5	0.00	0.01	0.0	0.21	0.09	0.18	0.02	0.03	0.01	0.01
3	Neem deoiled cake	0.19	5.78	0.08	17.9	0.02	0.05	0.19	0.09	0.11	0.14	0.18	0.46	0.07	0.06
4	Castor deoiled cake	0.16	7.91	0.15	11.2	0.04	0.03	0.28	0.23	0.35	0.20	0.05	0.13	0.00	0.00
5	Sesame deoiled cake	0.19	10.4	0.14	16.8	0.07	010	0.01	0.14	0.11	0.17	0.02	0.01	0.02	0.01
6	Cotton deoiled cake	0.16	9.91	0.15	12.8	0.05	0.03	0.16	0.11	0.33	0.22	0.03	0.06	0.01	0.00
7	Coconut deoiled cake	0.14	8.84	0.17	12.0	0.00	0.01	0.16	0.04	0.20	0.17	0.09	0.23	0.12	0.01
8	Palm deoiled cake	0.16	7.20	0.13	11.4	0.01	0.01	0.06	0.18	0.1	0.13	0.16	0.18	0.01	0.01
9	Sunflower deoiled cake	0.18	12.4	0.10	10.6	0.07	0.02	0.11	0.22	0.08	0.31	0.02	0.01	0.01	0.02

[a] Screening conditions: 30 °C, 100 mL/min, 3 g, 60 min

Table S4. Physicochemical characteristics and adsorption performance of screened sorbents

S. No	Name of the biochar	CO_2[a] uptake (mmol/g)	S_{BET}[b] (m^2/g)	V_t[c] (cm^3/g)	V_{micro}[b] (cm^3/g)	V_{meso}[e] (cm^3/g)	CHNS (elemental percentage- %)					Atomic ratio	
							C	H	N	S	O	H/C	O/C
1	Ma-5-2	0.46	203	0.15	0.02	0.12	56.36	2.39	5.15	0	12.2	0.51	0.16
2	M-5-2	0.61	291	0.21	0.05	0.15	64.98	2.30	5.58	0.26	9.93	0.43	0.11
3	M-6-2	0.71	463	0.30	0.18	0.12	66.79	2.16	5.29	0.21	9.22	0.39	0.10
4	M-7-2	0.65	351	0.23	0.08	0.14	62.80	1.81	4.18	0.12	8.62	0.35	0.10
5	M-6-1.5	0.66	327	0.21	0.07	0.13	63.79	2.26	5.31	0.28	11.8	0.43	0.14
6	M-6-2.5	0.67	349	0.26	0.11	0.14	55.17	1.8	5.01	0.10	5.95	0.39	0.08
7	MT	0.82	559	0.32	0.21	0.11	61.22	1.89	16.2	4.01	8.54	0.39	0.10
8	MK	0.85	756	0.37	0.29	0.08	61.93	1.91	4.89	0.19	8.88	0.41	0.08
9	MTKS	1.09	1031	0.50	0.36	0.13	60.60	2.48	7.92	1.18	6.73	0.49	0.08
10	MTKI	1.16	1304	0.52	0.44	0.07	60.84	1.9	6.21	1.07	6.83	0.37	0.07
11	MTKH	1.26	2420	0.67	0.55	0.17	61.65	1.1	10.34	3.19	7.52	0.21	0.13
12	MTKH-1	1.14	1769	0.63	0.49	0.13	64.64	1.5	10.6	3.34	5.95	0.28	0.12
13	MTKH-2	1.26	2420	0.67	0.55	0.17	61.65	1.1	10.34	3.19	7.52	0.21	0.13
14	MTKH-3	1.2	1860	0.62	0.47	0.15	65.70	1.6	10.06	2.6	7.98	0.29	0.11
15	MTKH-4	1.17	2156	0.65	0.53	0.12	67.21	1.3	7.8	2.19	7.62	0.23	0.09
16	MTKH-5	1.19	2198	0.65	0.52	0.14	62.06	1.6	11.49	3.68	6.97	0.31	0.14
17	MTKH-2-5-2	1.26	1904	0.66	0.42	0.24	60.75	1.75	11.61	3.54	8.01	0.34	0.09
18	MTKH-2-6-2	1.56	2996	0.69	0.62	0.09	62.07	1.42	13.2	3.53	7.89	0.27	0.09
19	MTKH-2-7-2	1.26	2420	0.67	0.55	0.12	61.65	1.1	10.34	3.19	7.52	0.21	0.13
20	MTKH-2-6-1.5	1.5	2212	0.65	0.54	0.11	62.94	2.06	11.28	3.61	7.73	0.39	0.09
21	MTKH-2-6-2.5	1.55	1958	0.62	0.51	0.10	64.67	1.47	11.1	3.58	7.80	0.27	0.09

[a] Screening conditions: 30 °C, 100 mL/min, 3 g, 60 min

[b] Surface area was calculated using the BET method at P/P_0=0.0-0.1

[c] Total pore volume at P/P_0=0.99

[d] Total micropore volume evaluated by the t-plot method.

[e] Total mesopore volume evaluated by the BJH-plot method.

Table S5. CO_2 capture results based on the CCD model in RSM

S No	A: Temp (°C)	B: Flowrate (mL/min)	C: Amount (g)	D: Time (min)	CO_2 uptake (mmol/g)
1	45	150	10.5	120	0.97
2	60	100	8	180	1.25
3	45	150	5.5	120	1.78
4	30	200	8	60	1.1
5	60	100	8	60	0.65
6	45	150	5.5	120	1.81
7	60	200	8	60	0.62
8	60	200	8	180	1.75
9	75	150	5.5	120	1.21
10	30	100	3	60	1.56
11	30	200	3	60	1.78
12	30	100	8	180	1.48
13	45	150	3	120	2.65
14	30	200	8	180	1.92
15	30	100	8	60	0.39
16	45	150	5.5	120	1.79
17	45	150	5.5	120	1.8
18	45	150	5.5	240	2.73
19	45	150	5.5	30	0.65
20	60	100	3	60	1
21	60	200	3	180	3.24
22	45	200	5.5	120	1.32
23	30	100	3	180	3.9
24	60	200	3	60	1.41
25	45	150	5.5	120	1.78
26	45	150	5.5	120	1.8
27	45	50	5.5	120	0.9
28	30	150	5.5	120	1.5
29	30	200	3	180	4.9
30	60	100	3	180	2.92

Table S6. ANOVA Quadratic table

Source	Sum of Squares	df	Mean Square	F-value	p-value	
Model	3.52	14	0.2516	29.86	< 0.0001	significant
A-Temp	0.1167	1	0.1167	13.85	0.0020	
B-Flow	0.1186	1	0.1186	14.08	0.0019	
C-Amount	1.35	1	1.35	160.56	< 0.0001	
D-Time	1.69	1	1.69	200.65	< 0.0001	
AB	0.0128	1	0.0128	1.52	0.2366	
AC	0.0435	1	0.0435	5.17	0.0382	
AD	0.0083	1	0.0083	0.9911	0.3353	
BC	0.0020	1	0.0020	0.2321	0.6370	
BD	0.0000	1	0.0000	0.0049	0.9449	
CD	0.0827	1	0.0827	9.82	0.0068	
A^2	0.0028	1	0.0028	0.3371	0.5701	
B^2	0.0112	1	0.0112	1.33	0.2662	
C^2	0.1252	1	0.1252	14.86	0.0016	
D^2	0.0060	1	0.0060	0.7102	0.4126	
Residual	0.1264	15	0.0084			
Lack of Fit	0.0968	10	0.0097	1.64	0.3058	not significant
Pure Error	0.0296	5	0.0059			
Cor Total	3.65	29				

Table S7: Fit statistics and models

Adsorbent	R^2	Adjusted R^2	Predicted R^2	Std. dev.	CV%	Model
MTKH-2-6-2	0.9654	0.9330	0.8185	0.0918	7.29	Quadratic

Table S8. Various kinetic model parameters for CO_2 adsorption

S. No	Models	Parameters
1	Pseudo-First Order	Q_e (exp) = 6.6 mmol. g^{-1}<br>Q_e (fit) = 4.01mmol. g^{-1}<br>K_1 = 0.216 h^{-1}<br>R^2 = 0.93
2	Pseudo-Second Order	Q_e (exp) = 6.6 mmol. g^{-1}<br>Q_e (fit) = 6.11 mmol. g^{-1}<br>K_2 = 0.092 g. $mmol^{-1}$. h^{-1}<br>R^2 = 0.98

3	Elovich	α =8.71 mmol. g^{-1}. h^{-1}<br>β = 0.627 g. $mmol^{-1}$<br>R^2 = 0.83
4	Weber-Morris	K_d =1.451 mmol. g^{-1}. $h^{-0.5}$<br>C = 1.906 mmol. g^{-1}<br>R^2 = 0.92

Table S9. Isotherm model parameters for CO_2 adsorption

S. No	Models	Parameters
1	Langmuir	q_m= 7.16 mmol g^{-1}<br>k_L= 97 atm^{-1}<br>R^2=0.99
2	Freundlich	K_F=7.19 mmol g^{-1} $atm^{-1/n}$<br>n=27.93<br>R^2=0.95

Coded Equation:

CO_2 Uptake = 1.23 - (0.0792*A) + (0.0799*B) – (0.2697*C) + (0.2815*D) – (0.0283*AB) + (0.0522*AC) – (0.0228*AD) + (0.0111*BC) + (0.0016*BD) – (0.0719*CD) + (0.0133*A^2) – (0.0265*B^2) + (0.0884*C^2) – (0.0175*D^2)

Actual Equation:

CO_2 Uptake = 1.21526 – (0.009550*Temperature) + (0.005921*Flow rate) – (0.281743*Amount of loading) + (0.009555*time) – (0.000038*Temperature *Flow rate) + (0.001391*Temperature*Amount of loading) – (0.000025*Flow rate*Amount of loading) + (5.37429E-07*Flow rate * time) – (0.000479*Amount*time) + (0.000059*Temperature^2) – (0.000059*Flow rate^2) + (0.014141*Amount of loading^2) – (4.85304E-06*time^2)

References:

[1] N. Das, P.K. Jena, D. Padhi, M. Kumar Mohanty, G. Sahoo, A comprehensive review of characterization, pretreatment and its applications on different lignocellulosic biomass for bioethanol production, Biomass Convers Biorefin 13 (2023) 1503–1527. https://doi.org/10.1007/s13399-021-01294-3.

[2] M. Kumar, S.N. Upadhyay, P.K. Mishra, A comparative study of thermochemical characteristics of lignocellulosic biomasses, Bioresour Technol Rep 8 (2019) 100186. https://doi.org/10.1016/J.BITEB.2019.100186.

[3] J. Cai, Y. He, X. Yu, S.W. Banks, Y. Yang, X. Zhang, Y. Yu, R. Liu, A. V. Bridgwater, Review of physicochemical properties and analytical characterization of lignocellulosic biomass, Renewable and Sustainable Energy Reviews 76 (2017) 309–322. https://doi.org/10.1016/J.RSER.2017.03.072.

[4] D.P. Maurya, A. Singla, S. Negi, An overview of key pretreatment processes for biological conversion of lignocellulosic biomass to bioethanol, 3 Biotech 5 (2015) 597–609. https://doi.org/10.1007/s13205-015-0279-4.

[5] M.O. Fajobi, O.A. Lasode, A.A. Adeleke, P.P. Ikubanni, A.O. Balogun, Investigation of physicochemical characteristics of selected lignocellulose biomass, Sci Rep 12 (2022). https://doi.org/10.1038/s41598-022-07061-2.

[6] L. Rajpoot, A. Tagade, G. Deshpande, K. Verma, S.R. Geed, D.S. Patle, A.N. Sawarkar, An overview of pyrolysis of de-oiled cakes for the production of biochar, bio-oil, and pyro-gas: Current status, challenges, and future perspective, Bioresour Technol Rep 19 (2022) 101205. https://doi.org/10.1016/J.BITEB.2022.101205.

[7] R. Kumar Singh, T. Patil, D. Pandey, A.N. Sawarkar, Pyrolysis of mustard oil residue: A kinetic and thermodynamic study, Bioresour Technol 339 (2021) 125631. https://doi.org/10.1016/J.BIORTECH.2021.125631.

[8] R. Rani, L.S. Badwaik, Functional Properties of Oilseed Cakes and Defatted Meals of Mustard, Soybean and Flaxseed, Waste Biomass Valorization 12 (2021) 5639–5647. https://doi.org/10.1007/s12649-021-01407-z.

[9] A. Gupta, R. Chaudhary, S. Sharma, Potential applications of mahua (Madhuca indica) biomass, Waste Biomass Valorization 3 (2012) 175–189. https://doi.org/10.1007/s12649-012-9107-9.

[10] H. V Mulimani, M.C. Navindgi, High Calorific Value Fuel from Pyrolysis of Waste De-Oiled Seed Cakes, n.d. www.neptjournal.com.

[11] H. V. Mulimani, M.C. Navindgi, Production and characterization of bio-oil by pyrolysis of mahua de-oiled seed cake, ChemistrySelect 3 (2018) 1102–1107. https://doi.org/10.1002/slct.201702198.

[12] A.K. Biswal, C. Lenka, P.K. Panda, J.M. Yang, P.K. Misra, Investigation of the functional and thermal properties of Mahua deoiled cake flour and its protein isolate for prospective food applications, LWT 137 (2021) 110459. https://doi.org/10.1016/J.LWT.2020.110459.

[13] A.K. Rajak, M. Harikrishna, S.F. Zeenath, S. Dalal, M.S.L. Karuna, K. Rajesh, R. Pothu, V. Vennu, R. Boddula, K. V. Padmaja, Transesterification of neem seed oil for environmentally friendly biolubricants: Promoting circular economy in industrial processes, Biomass Bioenergy 200 (2025) 108012. https://doi.org/10.1016/J.BIOMBIOE.2025.108012.

[14] M. Deshmukh, A. Pande, A. Marathe, Different particle size study of castor deoiled cake for biofuel production with an environmental sustainability perspective, Heliyon 8 (2022) e09710. https://doi.org/10.1016/J.HELIYON.2022.E09710.

[15] L.M. Ferreira, R. Rodolfo De Melo, A. Santos Pimenta, T. Kelly Barbosa De Azevedo, C. Brito De Souza, Adsorption performance of activated charcoal from castor seed cake prepared by chemical activation with phosphoric acid, (n.d.). https://doi.org/10.1007/s13399-020-00660-x/Published.

[16] R.Y. Zhang, H.M. Liu, J. Hou, Y.G. Yao, Y.X. Ma, X. De Wang, Cellulose fibers extracted from sesame hull using subcritical water as a pretreatment, Arabian Journal of Chemistry 14 (2021) 103178. https://doi.org/10.1016/J.ARABJC.2021.103178.

[17] S. Ramachandran, S.K. Singh, C. Larroche, C.R. Soccol, A. Pandey, Oil cakes and their biotechnological applications – A review, Bioresour Technol 98 (2007) 2000–2009. https://doi.org/10.1016/J.BIORTECH.2006.08.002.

[18] M.A. Sokoto, B. Biswas, J. Kumar, T. Bhaskar, Slow pyrolysis of Defatted Seeds Cakes of African star apple and silk cotton for production of bio-oil, Renew Energy 146 (2020) 1710–1716. https://doi.org/10.1016/J.RENENE.2019.07.145.

[19] A. Dukare, R. Yadav, S. Kautkar, P. Kuppusamy, K. Sharma, A. Shaikh, A. Pawar, A. Gadade, N. Vigneshwaran, S. Saxena, S.K. Shukla, Waste to wealth: Microbial-based sustainable valorization of cotton biomass, processing waste and by-products for bioenergy and other value-added products to promote circular economy, Waste Management Bulletin 2 (2024) 262–280. https://doi.org/10.1016/J.WMB.2024.11.011.

[20] R. Leesing, T. Somdee, S. Siwina, Y. Ngernyen, K. Fiala, Production of 2G and 3G biodiesel, yeast oil, and sulfonated carbon catalyst from waste coconut meal: An integrated cascade biorefinery approach, Renew Energy 199 (2022) 1093–1104. https://doi.org/10.1016/J.RENENE.2022.09.052.

[21] Z. Fu, L. Zhong, Y. Tian, X. Bai, J. Liu, Identification of Cellulose-Degrading Bacteria and Assessment of Their Potential Value for the Production of Bioethanol from Coconut Oil Cake Waste, Microorganisms 12 (2024). https://doi.org/10.3390/microorganisms12020240.

[22] S. Mi, H. Li, S. Li, Y. Han, The synergism of hot water pretreatment and enzymatic hydrolysis in depolymerization of lignocellulosic content of palm kernel cake, J Mol Catal B Enzym 134 (2016) 37–42. https://doi.org/10.1016/J.MOLCATB.2016.09.004.

[23] S. Kanchanasuta, N. Pisutpaisal, Improvement of glycerol waste utilization by co-feedstock with palm oil decanter cake on biohydrogen fermentation, Int J Hydrogen Energy 42 (2017) 3447–3453. https://doi.org/10.1016/J.IJHYDENE.2016.12.134.

[24] R.P. Kaur, G. Ghoshal, Sunflower protein isolates-composition, extraction and functional properties, Adv Colloid Interface Sci 306 (2022) 102725. https://doi.org/10.1016/J.CIS.2022.102725.

[25] J. Bautista, J. Parrado, A. Machado, Composition and Fraetionation of Sunflower Meal: Use of the Lignocellulosic Fraction as Substrate in Solid-State Fermentation, 1990.

[26] W. Wu, C. Wu, J. Liu, H. Yan, G. Zhang, G. Li, Y. Zhao, Y. Wang, Nitrogen-doped porous carbon through K2CO3-activated bamboo shoot shell for an efficient CO2 adsorption, Fuel 363 (2024). https://doi.org/10.1016/j.fuel.2024.130937.

[27] H. Li, M. Tang, X. Huang, L. Wang, Q. Liu, S. Lu, An efficient biochar adsorbent for CO2 capture: Combined experimental and theoretical study on the promotion mechanism of N-doping, Chemical Engineering Journal 466 (2023) 143095. https://doi.org/10.1016/J.CEJ.2023.143095.

[28] S. He, G. Chen, H. Xiao, G. Shi, C. Ruan, Y. Ma, H. Dai, B. Yuan, X. Chen, X. Yang, Facile preparation of N-doped activated carbon produced from rice husk for CO2 capture, J Colloid Interface Sci 582 (2021) 90–101. https://doi.org/10.1016/J.JCIS.2020.08.021.

[29] J. Han, L. Zhang, B. Zhao, L. Qin, Y. Wang, F. Xing, The N-doped activated carbon derived from sugarcane bagasse for CO2 adsorption, Ind Crops Prod 128 (2019) 290–297. https://doi.org/10.1016/j.indcrop.2018.11.028.

[30] J. Chen, J. Yang, G. Hu, X. Hu, Z. Li, S. Shen, M. Radosz, M. Fan, Enhanced CO2 Capture Capacity of Nitrogen-Doped Biomass-Derived Porous Carbons, ACS Sustain Chem Eng 4 (2016) 1439–1445. https://doi.org/10.1021/acssuschemeng.5b01425.

[31] S.M. Varghese, A.R. Chowdhury, D.N. Arnepalli, G. Ranga Rao, Delineating the effects of pore structure and N-doping on CO2 adsorption using coco peat derived carbon, Carbon Trends 10 (2023) 100250. https://doi.org/10.1016/J.CARTRE.2023.100250.

[32] C. Ma, J. Bai, M. Demir, X. Hu, S. Liu, L. Wang, Water chestnut shell-derived N/S-doped porous carbons and their applications in CO2 adsorption and supercapacitor, Fuel 326 (2022) 125119. https://doi.org/10.1016/J.FUEL.2022.125119.

[33] G. Nazir, A. Rehman, S.J. Park, Role of heteroatoms (nitrogen and sulfur)-dual doped corn-starch based porous carbons for selective CO2 adsorption and separation, Journal of CO2 Utilization 51 (2021) 101641. https://doi.org/10.1016/J.JCOU.2021.101641.

[34] J. Luo, B. Liu, R. Shi, Y. Guo, Q. Feng, Z. Liu, L. Li, K. Norinaga, The effects of nitrogen functional groups and narrow micropore sizes on CO2 adsorption onto N-doped biomass-based porous carbon under different pressure, Microporous and Mesoporous Materials 327 (2021) 111404. https://doi.org/10.1016/J.MICROMESO.2021.111404.
